# Truncated Equinin B Variants Reveal the Sequence Determinants of Antimicrobial Selectivity

**DOI:** 10.3390/md24010046

**Published:** 2026-01-17

**Authors:** Mariele Staropoli, Theresa Schwaiger, Jasmina Tuzlak, Renata Biba, Lukas Petrowitsch, Johannes Fessler, Marin Roje, Matteo Cammarata, Nermina Malanović, Andreja Jakas

**Affiliations:** 1 Laboratory for Chiral Technologies, Division of Organic Chemistry and Biochemistry, Ruđer Bošković Institute, Bijenička cesta 54, 10000 Zagreb, Croatia; mariele.staropoli@unipa.it (M.S.); marin.roje@irb.hr (M.R.); 2 Department of Earth and Marine Sciences, University of Palermo, Viale delle Scienze, Ed. 16, 90128 Palermo, Italy; matteo.cammarata@unipa.it; 3 Institute of Molecular Bioscience, University of Graz, Humboldstr. 50, 8010 Graz, Austria; theresa.schwaiger@uni-graz.at (T.S.); jasmina.tuzlak@uni-graz.at (J.T.); lukas.petrowitsch@uni-graz.at (L.P.); 4 Division of Molecular Medicine, Ruđer Bošković Institute, Bijenička cesta 54, 10000 Zagreb, Croatia; renata.biba@irb.hr; 5 Division of Immunology, Otto Loewi Research Center, Medical University of Graz, 8036 Graz, Austria; 6 Field of Excellence BioHealth, University of Graz, 8010 Graz, Austria; 7 Bio TechMed Graz, 8010 Graz, Austria

**Keywords:** marine peptides, solid phase peptide synthesis, structure-function relationship, membrane permeability, peptide specificity, peptide–membrane interactions, Gram-positive bacteria

## Abstract

Equinin B (GQCQRKCLGHCSKKCPKHPQCRKRCIRRCFGYCL), a marine peptide from *Actinia equina* exhibits antibacterial activity against both Gram-positive and Gram-negative bacteria. To identify a smaller active region and explore tunable properties, three peptide fragments were synthesized: GQCQRKCLGHCS (**EB1**), KKCPKHPQCRK (**EB2**), and RCIRRCFGYCL (**EB3**), yielding peptides with key AMP-like properties, including the most positively charged and most hydrophobic regions. Only the 11-residue C-terminal fragment showed selective activity against Gram-positive bacteria, including *Staphylococcus epidermidis*, *Bacillus subtilis*, and *Enterococcus hirae*, while remaining inactive against *Escherichia coli*. Peptide modifications, achieved by replacing cysteine residues with arginine, generally did not enhance activity, but in the C-terminal fragment **EB3** they reduced hemolytic activity and increased bacterial specificity. Membrane depolarization assays confirmed that the unmodified fragment **EB3** strongly compromises bacterial membranes, whereas the modified variant showed minimal depolarization, highlighting its markedly reduced membrane-perturbing potential. *In silico* modelling revealed that the **EB3** can adopt multiple membrane-disruption modes, from transient shallow pores to carpet-like mechanisms, while the cysteine-to-arginine variant interacts mainly via partial insertion anchored by arginine residues. Phenylalanine appears to interact with the membrane, and reducing hydrophobicity by its removal abolished antibacterial activity. These findings highlight the 11-residue C-terminal fragment as a tunable, membrane-targeting motif with mechanistic novelty, offering a blueprint for developing safer, selective antimicrobial peptides with reduced cytotoxicity.

## 1. Introduction

Even though the discovery of antibiotics revolutionized medicine, their inappropriate use has enabled the spread of resistant bacteria and rendered commercially available antibiotics ineffective. Antibiotic resistance (AR) is one of the greatest threats, and the development of new molecules with antibacterial activity has become a global priority [1,2,3]. Antimicrobial peptides (AMPs), naturally occurring defense molecules found across many species, represent a powerful alternative antibacterial strategy against antibiotic-resistant pathogens [4]. As part of the innate immune response, they have low antigenicity and exhibit antimicrobial activity against Gram-positive and Gram-negative bacteria, fungi, viruses, and parasites. Because AMPs have multiple mechanisms of action, they have a low potential for inducing resistance [5,6,7]. Terrestrial environments have been primarily studied for natural peptides useful to the pharmaceutical industry, even though the marine environment covers 70% of the earth’s surface [8,9]. Only in recent decades has the proven therapeutic and anti-infective potential of peptides from marine sources opened a wide field of interest for researchers and companies seeking valuable bioactive compounds from marine fauna and flora [10]. Peptides derived from marine sources have different functions and structures than those isolated from terrestrial sources, due to the distinct adaptive pressures these organisms have experienced during evolution. Most bioactive compounds isolated from marine environments originate from symbiotic microorganisms and organisms, especially mollusks and cnidarians [11,12]. One of these is *Actinia equina* (Linnaeus, 1758), a little-studied anthozoan coelenterate of the *Actiniidae* family, widespread from the Indo-Pacific to the Mediterranean and the Atlantic. It is exposed to harsh conditions since it lives mainly in intertidal zones but also up to a few meters deep, and has developed the capability to remain out of the sea for hours [13]. From this cnidarian’s tentacle, two peptides called equinin A and B were isolated [13]. Equinin B (EB) showed promising antibacterial activity (minimum inhibitory concentrations (MICs) and bactericidal concentrations of 1 mg/mL and 0.25 mg/mL, respectively). The amino acid sequence GQCQRKCLGHCSKKCPKHPQCRKRCIRRCFGYCL, with 34 amino acids, shows a similarity of 37.78% with aurelin, a 40-residue AMP isolated from the mesoglea of the jellyfish *Aurelia aurita* which exhibits activity against Gram-positive and Gram-negative bacteria [14]. Equinin B is highly cationic with multiple positively charged residues and cysteines, giving it an overall strong net positive charge characteristic of antimicrobial peptides that interact with bacterial membranes. *In silico* structural modeling using SWISS-MODEL based on similarity to the aurelin suggests that the peptide may adopt an α-helical conformation as part of its three-dimensional structure, a typical feature seen in many small AMPs that facilitates amphipathic membrane binding and disruption [15]. The structure of equinin B is not fully resolved. While it contains cysteines that could form disulfide bridges and loops, it is not confirmed to be cyclic. Published models suggest a partially α-helical structure, although the reliability of the model is low. The complex globular two-helix bundle of equinin B motivated the design of shorter, linear peptide fragments that retain essential functional properties. It was also found that truncated peptides derived from aurelin and other AMPs could have significant antimicrobial activity [16,17]. The advantage of peptide-based drugs is that they can be easily designed and rapidly synthesized with optimal purity and high yield [18]. This is possible thanks to the development of solid-phase peptide synthesis (SPPS) by Merrifield in 1963 [19]. This methodology has been improved and automated over the years, allowing the preparation of peptide sequences in large quantities and within short timescales [20]. The synthesis of smaller peptides is easier and faster than that of longer peptides, and truncated parts of bioactive peptides that still retain activity are valuable.

**Figure 1:**
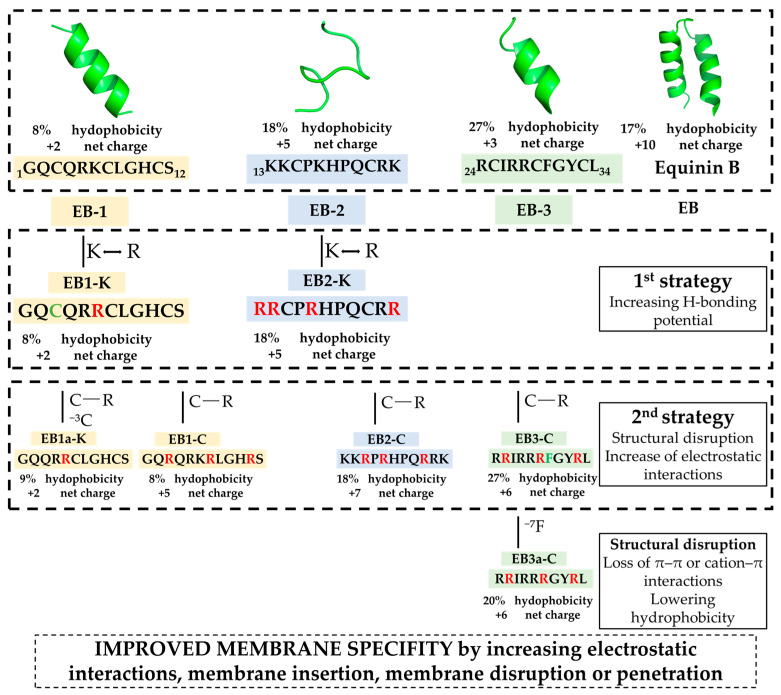
Schematic representation of the design strategy. Structure were predicted with PepFold3 [21].

Herein, we fragmented equinin B sequence into three parts: the first part contains 12 residues, and the second and third parts contain 11 residues each: GQCQRKCLGHCS (**EB1**), KKCPKHPQCRK (**EB2**) and RCIRRCFGYCL (**EB3**), respectively. In the second series, all Lys residues (if present in the peptide) were replaced by Arg (**EB1-K** and **EB2-K**), and in a third series, all Cys residues were replaced by Arg (**EB1-C**, **EB2-C** and **EB3-C**) to enhance membrane interactions and prevent intra- or intermolecular disulfide bond formation. (Figure 1). Some amino acids were removed from the peptides, resulting in **EB1a-K** (C3) and **EB3a-C** (F7)**.** After evaluating the antimicrobial activity against *Escherichia coli* and *Staphylococcus epidermidis*, two active peptides, **EB3** and **EB3-C**, were identified. **EB3** displayed broad, non-specific activity, whereas **EB3-C** was active only against bacteria and showed no hemolytic effects. Their distinct activities appear to arise from structural differences, with **EB3-C** likely inducing only small membrane defects that selectively destabilize bacterial membranes.

## 2. Results and Discussion

### 2.1. Structure-Function Guided Design of the Peptide Library

To identify biologically active regions in the equinin B sequence, we generated a set of peptides and applied structure–function–driven modifications to enhance their antimicrobial activity, as shown in Figure 1. In the initial set of peptide syntheses, we prepared three truncated peptides, **EB** (**1**–**3**), derived from EB (Figure 1). The residues are identical to those in equinin B, but the sequences comprised: the first 12 residues, the residues from 13–23 and 24–34, resulting in three peptides, **EB1**, **EB2** and **EB3** (Figure 1). This strategy enabled direct assessment of the antimicrobial contributions of the most positively charged versus the most hydrophobic regions, which are considered key determinants of antimicrobial activity in peptides. We first evaluated their antimicrobial activity against *Escherichia coli* and *Staphylococcus epidermidis* over a concentration range of 0.8 to 100 µM (Table 1). Determination of their MICs in cation-adjusted Mueller–Hinton broth (MHB) revealed that none of the peptides exhibited inhibitory activity against *E. coli* or *S. epidermidis* within this range, except for peptide **EB3**, which had a MIC of 25 µM against *S. epidermidis*. Assuming the **EB3** region is associated with antimicrobial activity, we analyzed its amino acid composition. Since it lacked lysines, we replaced all lysines in **EB1** and **EB2** with arginine, a modification expected to enhance hydrogen-bonding capacity and strengthen interactions with membrane phospholipids, thereby improving membrane-disruptive activity—a hallmark of classical antimicrobial peptides [22]. This step led to the generation of the modified peptides **EB1-K** and **EB2-K**. However, this modification did not improve activity against either *E. coli* or *S. epidermidis*. To further enhance membrane interactions, we replaced all cysteines with arginines in all three peptides. Cysteines can form disulfide bridges, which may constrain peptide flexibility and affect membrane interactions; replacing them with arginine was expected to increase positive charge, reduce disulfide bond formation, and enhance peptide–membrane interactions. Despite these modifications, the antimicrobial activity of all peptides remained unchanged. Nevertheless, only **EB3-C** retained antimicrobial activity, and this activity was observed solely against *S. epidermidis*. We also investigated whether removing the cysteine at position 3 in **EB1-K** would affect activity, resulting in the peptide **EB1a-K**; however, this modification did not improve antimicrobial activity. Notably, the **EB3** peptide had the highest hydrophobic content at 27%, whereas all other peptides ranged between 8% and 18%. To evaluate whether hydrophobicity contributes to antimicrobial activity, we removed the phenylalanine at position 7 in **EB3-C**, generating the peptide **EB3a-C**. This modification abolished activity against *S. epidermidis,* confirming that hydrophobicity is likely the driving force behind the antimicrobial activity of **EB3**.

Peptides were synthesized using an automated Fmoc-SPPS approach from the C-terminus to the N-terminus.

The peptides were synthesized on Wang resin with the C-terminal amino acid attached as follows: Fmoc-Ser-Wang resin, Fmoc-Lys(Boc)-Wang resin, Fmoc-Arg(Pbf)-Wang resin, and Fmoc-Leu-Wang resin (Table S1). Synthesis was performed in dimethylformamide using HBTU/OxymaPure^®^ as coupling reagents. The Fmoc group from the first amino acid and subsequent residues after coupling was removed with 20% piperidine in DMF. For activation of carbonyl groups, 0.4 M *N*-methylmorpholine (NMM) was used. After completing the peptide sequence, the peptide was cleaved from the resin and the protecting groups were removed with 95:2.5:2.5 TFA:TIS:H_2_O. After deprotection, peptides were precipitated in diisopropyl ether and centrifuged. Following HPLC purification, the compounds were desalted on SPE C18 columns and lyophilized.

### 2.2. Evaluation of Peptide Specificity

**Figure 2:**
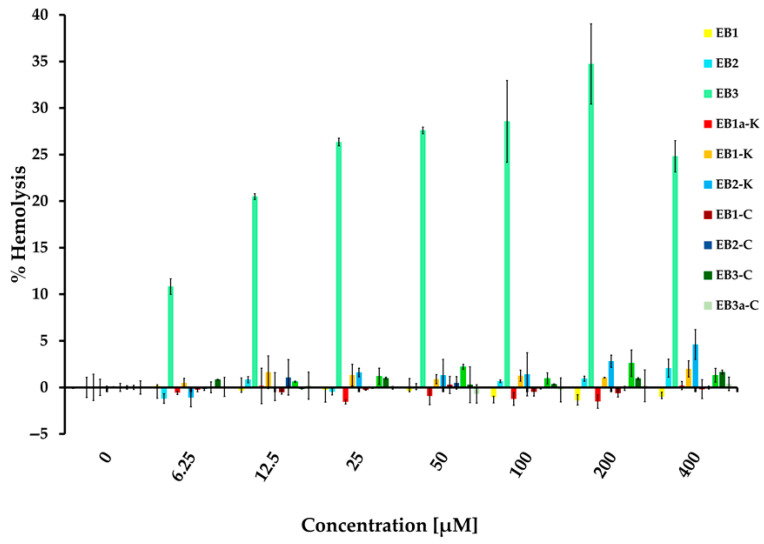
Hemolysis of peptides. Hemolytic activity of peptides was estimated in 2.5% human erythrocytes derived from pooled whole human blood of at least 10 donors.

To assess whether the peptides exhibit activity against other cell types, such as human erythrocytes, and to estimate their specificity and therapeutic index, we performed hemolysis assays (Figure 2). None of the peptides showed hemolytic activity up to 400 µM, except for **EB3**, which caused significant lysis at concentrations lower than its MIC. Interestingly, replacing all cysteines in **EB3** improved its cytotoxic profile, as **EB3-C** did not induce hemolysis. Although all truncated peptides from the first batch contain cysteine residues, only **EB3** showed antimicrobial activity, which was retained after cysteine removal, whereas hemolytic activity was lost. This suggests that cysteine contributes to eukaryotic cell interactions but is not required for antibacterial function. This modification resulted in a notable therapeutic index greater than 16, calculated as 400 µM (no hemolysis) divided by 25 µM (MIC against *S. epidermidis*).

### 2.3. In Silico Structural Profiling

**Figure 3:**
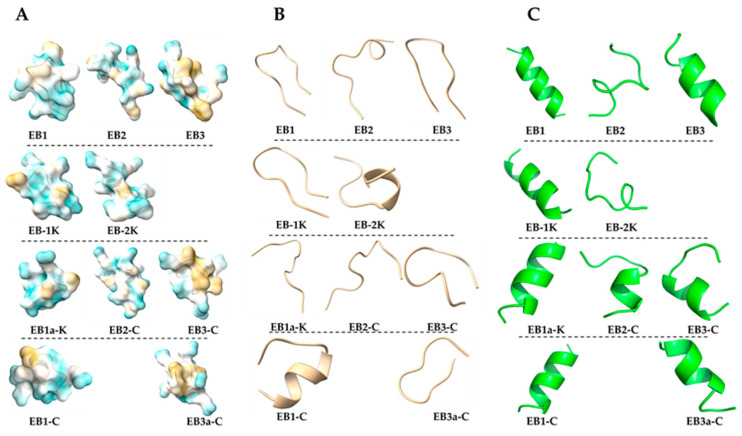
Structural surface characteristics of the conformational assemblies of peptides (**A**) AlphaFold predictions of secondary structure exposing charged (blue) and hydrophobic regions (beige). (**B**) AlphaFold predictions illustrating the folding topology. (**C**) PepFold predictions illustrating the folding topology.

*In silico* structural profiling revealed that most of the peptides have poorly defined secondary structures, with a general tendency toward alpha-helix formation in PepFold3 predictions, while AlphaFold3 predictions remained largely disordered without defined secondary conformations (Figure 3). Among them, **EB3** adopts the most structurally distinctive alpha-helix, featuring less flexible termini and a distinct segregation of charged and hydrophobic residues—hallmarks of an amphipathic helix. This structural arrangement is reflected in its high amphipathic character, as indicated by the highest hydrophobic moment (µ) among the peptides analyzed. Moreover, **EB3** exhibits the lowest ΔG for partitioning from water to octanol, suggesting a strong potential for membrane interaction (Table 2). Altogether, these features are consistent with key characteristics commonly observed in antimicrobial peptides [23,24]. High µ values are often associated with increased cytotoxicity toward eukaryotic membranes due to stronger and less selective membrane interactions. In this context, it is noteworthy that **EB3** exhibited hemolytic activity, whereas its analog **EB3C** did not. This suggests that reducing amphipathicity may enhance peptide specificity for bacterial membranes. Notably, the modification from **EB3** to **EB3-C** also led to a complete loss of α-helical structure when modeled with AlphaFold [25], while PepFold predicted only a small remaining α-helical segment. Whether this difference in secondary structure contributes to the observed increase in selectivity remains an open question. However, the larger, contiguous hydrophobic patch in the **EB3** peptide, compared with the more distributed hydrophobicity of **EB3-C** may explain why **EB3** is active against both bacteria and erythrocytes, whereas **EB3-C** remains selective for bacteria.

The bilayer partitioning free energy (ΔG) describes the energetic cost of transferring peptide residues from an aqueous environment into the hydrophobic core of a membrane. It is typically estimated using the water-to-octanol scale, which serves as an experimental proxy for membrane partitioning. Lower (more negative) ΔG values indicate a greater tendency for the peptide to insert into the lipid bilayer, promoting membrane association and potential disruption. Conversely, higher (less negative or positive) ΔG values suggest limited membrane affinity and lower antimicrobial potential [26]. Accordingly, the more favorable (negative) ΔG values observed for **EB3** and its analogs suggest a potential for stronger membrane activity compared to **EB1** and **EB2**.

### 2.4. Evaluation of Membrane Permeability and Activity

**Figure 4:**
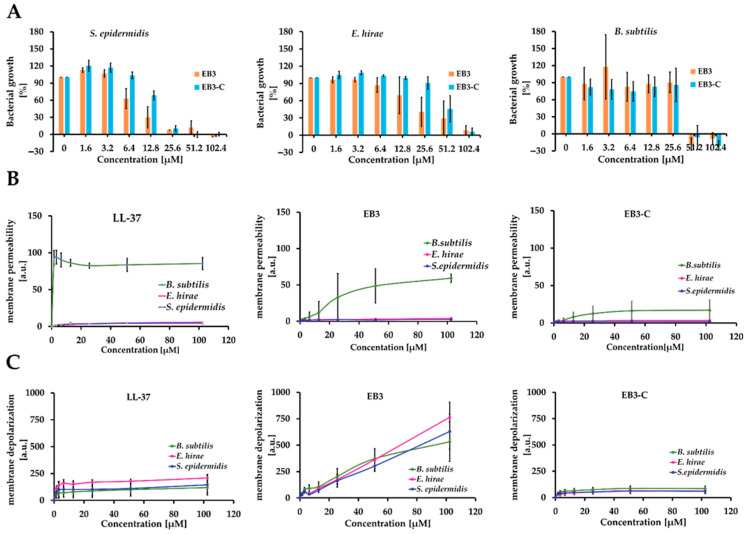
Impact on the Membrane Integrity by EB peptides. (**A**) Bacterial growth, (**B**) membrane permeability and (**C**) Membrane depolarization of *B. subtilis*, *E. hirae* and *S. epidermidis* exposed to **EB3** and **EB3-C**. Data of (**A**,**B**) are mean results of three independent experiments performed in duplicates. Data of (**C**) are mean results of two independent experiments performed in duplicates.

To further evaluate the antimicrobial activity and specificity of **EB3** and **EB3-C**, we determined their MIC values against *Bacillus subtilis* and *Enterococcus hirae*, using LL-37 as a reference peptide (Figure 4A). Although EB, a 34-amino-acid likely cyclic peptide, is active against both Gram-positive and Gram-negative bacteria at low concentrations [13], comparison with our short linear peptides is limited by differences in experimental conditions and the distinct structure, properties, and mode of action of cyclic peptides, particularly with respect to membrane disruption, where peptide length can lead to hydrophobic mismatch and different extents of membrane neutralization. Instead of EB, we used LL-37 as a control peptide because it is linear and its membrane-targeting mode of action is well characterized [27,28,29,30]. Both **EB3** and **EB3-C** exhibited antimicrobial activity against these strains. The MIC ranged from 25.6 to 102.4, showing the following order of activity for both peptides: *S. epidermidis* > *B. subtilis* > *E. hirae*. To investigate whether their mode of action involves membrane permeabilization, we performed propidium iodide (PI) uptake assays (Figure 4B), in which the fluorescent dye penetrates cells only when membrane integrity is compromised. While LL-37 caused extensive membrane permeabilization in *B. subtilis*, the EB peptides did not induce significant membrane damage even at concentrations up to 400 µM. A slight increase in permeability was observed for both EB peptides, representing only about 20% of the level induced by LL-37. None of the peptides permeabilized *E. hirae* or *S. epidermidis*. These results suggest that the EB peptides may act through a more specific, possibly non-lytic mechanism, and that *B. subtilis* may be more sensitive to their activity. However, since LL-37, a peptide with a well-established membrane-disruptive mechanism, was also unable to permeabilize *E. hirae* and *S. epidermidis*, it is likely that smaller or transient membrane perturbations, undetectable by PI staining, could still be sufficient to cause bacterial death. To test whether small defects in the membrane contribute to the antimicrobial effect, we performed a membrane depolarization assay using the voltage-sensitive dye DiSC_3_(5). The results showed that all peptides induced slight depolarization of the bacterial membrane, with **EB3** showing a markedly stronger signal than the other variants. These data support the idea that EB-3’s antimicrobial effect is closely tied to its ability to disrupt membrane potential. Although some degree of membrane depolarization is detectable for **EB3-C**, the effect is relatively modest, and it remains unclear whether **EB3-C** alters or depolarizes the membrane sufficiently for its antimicrobial activity to be directly attributed to membrane disruption. This pattern points toward a non-membranolytic or intracellular mode of action for **EB3-C**.

### 2.5. In Silico Evaluation of Peptide–Membrane Interactions

**Figure 5:**
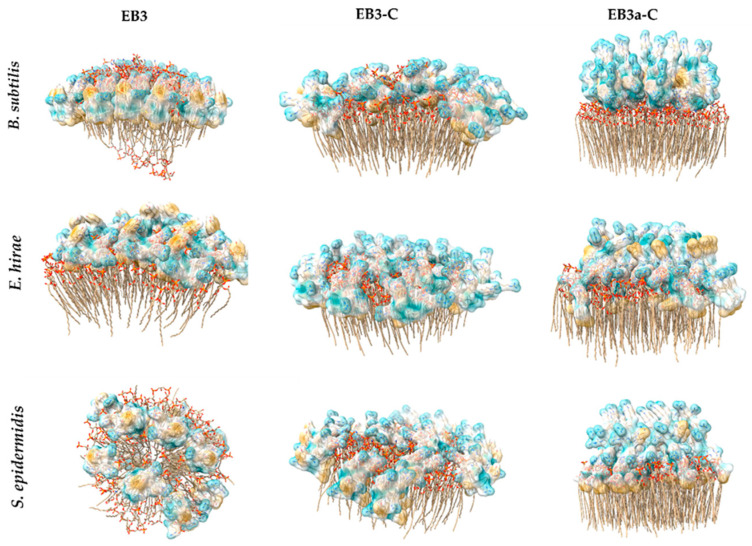
Prediction models of **EB3**, **EB3-C** and **EB3a-C** interacting with membranes mimicking *B. subtilis*, *E. hirae* and *S. epidermidis* cytoplasmic membranes.

To predict the molecular interactions of the peptides with bacterial membranes, we performed Alphafold modeling using membrane compositions resembling *B. subtilis*, *S.epidermidis* and *E. hirae* (Figure 5). For **EB3** interacting with *Bacillus* membranes, the peptide exhibited transient pore-like insertion accompanied by the formation of a short α-helix. Of note, AlphaFold3 predictions of the peptide in isolation indicated a lack of defined secondary structure, consistent with the notion that membrane interaction often induces secondary structure in antimicrobial peptides [31]. This insertion was relatively shallow, correlating with the limited membrane permeability observed experimentally. Notably, hydrogen bonds formed between the backbone of residues R1 and R5, where the N-terminal NH_3_ group interacts with the carbonyl oxygen of R5, along with similar interactions involving the amide nitrogen and residue C2. In contrast, interaction with *E. hirae* membranes showed peptide helix formation with surface association but no membrane insertion. Here, binding was primarily to phosphate (PO_4_) groups on the membrane surface, with residue Y9 oriented outward. For *S. epidermidis*, the peptide appeared to act via a detergent-like carpet mechanism, forming short aliphatic helices. Residue F7 inserted into the lipid bilayer along with L11 and I7, while R1 and R5 residues interacted with surface PO_4_ groups. Although no similar interactions were observed for R4, they remain possible. Consistent with *E. hirae*, Y9 faced outward, suggesting a conserved orientation.

**EB3-C** interacts with all Gram-positive membrane mimics through a consistent mechanism involving the formation of a short amphipathic helix, followed by membrane insertion primarily mediated by residue F7 and stabilized by interactions between R4/R6 and phosphate groups. In *B. subtilis* mimics, insertion occurs via F7 alone, whereas in *E. hirae* membranes both F7 and Y9 contribute to anchoring the peptide more deeply. For *S. epidermidis* mimics, insertion is supported by F7 together with L11, again combined with the characteristic R4/R6–phosphate interactions. These conserved electrostatic contacts, along with slight variations in hydrophobic anchoring residues, likely underpin **EB3-C**’s selective and non-hemolytic membrane-destabilizing activity.

Overall, the modeling indicates that **EB3** lacks membrane selectivity because it does not adopt a stable, deeply inserted conformation in any of the Gram-positive membrane mimics. Instead, it interacts in multiple, non-specific ways—ranging from shallow, transient insertion in *B. subtilis*, to purely surface binding in *E. hirae* and a carpet-like mode in *S. epidermidis*. This variability, along with its inconsistent hydrophobic anchoring and reliance on general phosphate interactions, prevents **EB3** from targeting a specific membrane architecture and explains its broad, non-selective activity. Interestingly, **EB3** also displays greater peptide–peptide interactions during membrane simulations, which along with its larger hydrophobic patch may underlie its reduced specificity and ability to disrupt both bacterial and erythrocyte membranes.

In addition, we performed structure predictions for **EB3a-C** to assess whether the F residue in **EB3-C** is important for membrane interaction. Modelling with *Bacillus* membranes revealed fish-hook-like structures on the surface, characterized by a backward-bent end forming a short β-sheet, with no deep membrane insertion. The peptide showed strong binding to phosphoglycolohydroxamic acid (PGH) on the membrane surface. For *E. hirae* and *S. epidermidis*, **EB3a-C** appeared mostly unfolded, with deep insertion of the C-terminal leucine (L10) into the membrane and association of arginine residues with phosphate (PO_4_) groups; notably, R1 was oriented outward. These observations suggest that **EB3a-C** interacts predominantly via surface binding and shallow insertion, relying on specific lipid interactions rather than deep membrane penetration. The structural differences from **EB3**, including β-sheet formation and altered insertion depth, likely contribute to its lack of activity.

## 3. Materials and Methods

### 3.1. General

Commercial reagents and solvents were obtained as follows: Fmoc-Ser(tBu)-Wang resin, Fmoc-Lys(Boc)-Wang resin, Fmoc-Leu-Wang resin, Fmoc-Cys(tBu)-OH, Fmoc-Gly-OH, Fmoc-Phe-OH, Fmoc-Lys(Boc)-OH, Fmoc-Leu-OH, Fmoc-Gln(Trt)-OH, Fmoc-Arg(Pbf)-OH, Fmoc-Tyr(tBu)-OH, Fmoc-Pro-OH and Fmoc-Ile-OH all purchased from Merck (Darmstadt, Germany).

NMR spectra were recorded on a Bruker AV 600 spectrometer, operating at 150.91 MHz for 13C and 600.13 MHz for 1H nuclei. The spectra were measured in deuterated dimethyl sulfoxide (DMSO-*d*_6_) at 25 °C. Chemical shifts in parts per million (ppm) were referenced to tetrametylsilane (TMS). Spectra were assigned based on one dimensional ^1^H, ^13^C and APT (Attached Proton Test) and 2D homonuclear COSY (Correlation Spectroscopy) and heteronuclear HMQC (Heteronuclear Multiple-Quantum Correlation) and HMBC (Heteronuclear Multiple Bond Correlation) experiments.

Solid phase peptide synthesis was carried out on SPPS PS3 (Protein Technologies, Inc., Tucson, AZ, USA) in dimethylformamide (DMF) as solvent, and 20% piperidine as deprotecting reagent and 0.4 M NMM as activating reagent in combination of coupling reagents HBTU and OximePure.

RP-HPLC analysis and purification was performed on analytical column Zorbax C18 (4.6 × 150 mm, 5 μm, Agilent Technologies, Waldbronn, Germany) and preparative column Zorbax (21.2 × 150 mm, 5 μm, Agilent Technologies, Waldbronn, Germany). The analyses were performed on an Agilent 1200 Series System (Agilent Technologies, Waldbronn, Germany), equipped with a vacuum degasser, a quaternary pump, a thermostated column compartment, an autosampler and a variable wavelength detector. The method used was gradient A as follows: 0.1% TFA/water (eluent 1); MeOH (eluent 2); 0–30 min 100% eluent 1 to 100% eluent 2; 30–35 min 100% eluent 2; 35–35.1 min 100% eluent 2 to 100% eluent 1; 35.1–40 min 100% eluent 1; flow rate: 1 mL min^−1^ (analytical) and 10 mL min^−1^ (preparative); detection wavelength: 210 and 215 nm; injection volume: 20–50 μL (analytical), 5 mL (preparative).

### 3.2. Peptide Synthesis

All peptides were synthesized on Wang resin by automated SPPS on a PS3 peptide synthesizer (Protein Technologies, Inc., Tucson, AZ, USA), following the Fmoc-strategy. The following Fmoc-amino acids were used: Fmoc-Ser(tBu)-Wang resin, Fmoc-Lys(Boc)-Wang resin, Fmoc-Leu-Wang resin, Fmoc-Cys(tBu)-OH, Fmoc-Gly-OH, Fmoc-Phe-OH, Fmoc-Lys(Boc)-OH, Fmoc-Leu-OH, Fmoc-Gln(Trt)-OH, Fmoc-Arg(Pbf)-OH, Fmoc-Tyr(tBu)-OH, Fmoc-Pro-OH and Fmoc-Ile-OH. Fmoc deprotections were performed with a solution of 20% piperidine in DMF. Peptide assembly was performed with the SPPS standard coupling cycle using commercial Wang resin with anchoring first (C-terminus) amino acid and following Fmoc-protected amino acids (4 eq in DMF), OxymaPure (4 equiv in DMF), and HBTU (4 equiv in DMF). Briefly, the resin charged with the all-protected Fmoc-peptide was treated with a mixture of TFA/TIS/H_2_O (95:2.5:2.5, *v*/*v*/*v*) at room temperature for cleavage from resin and deprotection of side amino acid chains. After 3 h, the resin was filtered off. The peptides were precipitated with cold diisopropyl ether, centrifuged and lyophilized. Purification and subsequent analysis of peptides was performed using HPLC as explained in Section 3.1. NMR spectra and assignation are shown in Supplementary Materials (SM): Figures S1–S20.

**EB-1**, Gly-Gln-Cys-Gln-Arg-Lys-Cys-Leu-Gly-His-Cys-Ser (GQCQRKCLGHCS)

Yield: 86%; C_50_H_86_N_20_O_18_S_3_. *t*_R_ (A) = 20.23 min. MS: *m*/*z* [M + H]^+^ (calc: 1318.58); [M + 2H]^2+^ calc. *m*/*z* 660.2925; exp. *m*/*z* 660.2924.

**EB-2**, Lys-Lys-Cys-Pro-Lys-His-Pro-Gln-Cys-Arg-Lys (KKCPKHPQCRK)

Yield: 77%; C_57_H_101_N_21_O_13_S_2_. *t*_R_ (A) = 14.52 min. MS: *m*/*z* [M + H]^+^ (calc: 1352.74); [M + 2H]^2+^ calc. *m*/*z* 676.8743; exp. *m*/*z* 676.8730.

**EB-3**, Arg-Cys-Ile-Arg-Arg-Cys-Phe-Gly-Tyr-Cys-Leu (RCIRRCFGYCL)

Yield: 77%; C_59_H_96_N_20_O_13_S_3_. *t*_R_ (A) = 23.34 minMS: *m*/*z* [M + H]^+^ (calc: 1389.67); [M + 2H]^2+^ calc. *m*/*z* 695.3392; exp. *m*/*z* 695.3413.

**EB1a-K**, Gly-Gln-Gln-Arg-Arg-Gly-His-Cys-Ser (GQQRRCLGHCS)

Yield: 55%; C_47_H_81_N_21_O_15_S_2_. *t*_R_ (A) = 18.23 min. MS: *m*/*z* [M + H]^+^ (calc: 1244.58) [M + 2H]^2+^ calc. *m*/*z* 622.7909; exp. *m*/*z* 622.7914.

**EB1-K**, Gly-Gln-Cys-Gln-Arg-Arg-Gly-His-Cys-Ser (GQCQRRCLGHCS)

Yield: 46%; C_50_H_86_N_22_O_16_S_3_. *t*_R_ (A) = 19.80 min. MS: *m*/*z* [M + H]^+^ (calc: 1244.58); [M + 2H]^2+^ calc. *m*/*z* 674.2955; exp. *m*/*z* 674.2955.

**EB2-K**, Arg-Arg-Cys-Pro-Arg-His-Pro-Gln-Cys-Arg-Arg (RRCPRHPQCRR)

Yield: 77%; C_57_H_101_N_29_O_13_S_2_. *t*_R_ (A) = 15.94 min MS: *m*/*z* [M + H]^+^ (calc: 1465.75); [M + 2H]^2+^ calc. *m*/*z* 732.8866; exp. *m*/*z* 732.8958.

**EB1-C**, Gly-Gln-Arg-Gln-Arg-Lys-Arg-Leu-Gly-His-Arg-Ser (GQRQRKRLGHRS)

Yield: 60%; C_59_H_107_N_29_O_16_. *t*_R_ (A) = 10.66 minMS: *m*/*z* [M + H]^+^ (calc: 1478.86); [M + 2H]^2+^ calc. *m*/*z* 739.9304; exp. *m*/*z* 739.9287.

**EB2-C**, Lys-Lys-Arg-Pro-Lys-His-Pro-Gln-Arg-Arg-Lys (KKRPKHPQRRK)

Yield: 52%; C_63_H_115_N_27_O_13_. *t*_R_ (A) = 9.53 min. MS: *m*/*z* [M + H]^+^ (calc: 1458.93); [M + 2H]^2+^ calc. *m*/*z* 729.9662; exp. *m*/*z* 729.9648.

**EB3-C**, Arg-Arg-Ile-Arg-Arg-Arg-Phe-Gly-Tyr-Arg-Leu (RRIRRRFGYRL)

Yield: 74%; C_68_H_117_N_29_O_13_. *t*_R_ (A) = 17.24 min. MS: *m*/*z* [M + H]^+^ (calc: 1548.95); [M + 2H]^2+^ calc. *m*/*z* 774.9771; exp. *m*/*z* 774.9764.

**EB3a-C**, Arg-Arg-Ile-Arg-Arg-Arg-Gly-Tyr-Arg-Leu (RRIRRRGYRL)

Yield: 24%; C_59_H_108_N_28_O_12_. *t*_R_ (A) = 15.70 min. MS: *m*/*z* [M + H]^+^ (calc: 1401.88); [M + 2H]^2+^ calc. *m*/*z* 701.4429; exp. *m*/*z* 701.4415.

### 3.3. Biological Activity

#### 3.3.1. Minimum Inhibitory Concentration (MIC) Assay

For these studies we used reference strains *Escherichia coli* ATCC25922, *Bacillus subtilis* 168c, *Staphylococcus epidermidis* DSM 20044 (DSMZ Gmbh, Brauschweig, Germany) and *Enterococcus hirae* DSM 3320 (DSMZ Gmbh, Germany). Bacterial cells were inoculated into Mueller–Hinton Broth II (MHB II; Sigma-Aldrich, Steinheim, Germany) and grown to the mid-logarithmic phase, washed in sodium buffered saline (PBS, 137 mM NaCl, 2.7 mM KCl, 10 mM Na_2_HPO_4_, and 2 mM KH_2_PO_4_, pH 7.4). The culture was adjusted to ~1 × 10^6^ CFU/mL and dispensed into 96-well microtiter plates. Peptides were tested in two-fold serial dilutions across a concentration range of 0.4–100 µM, each concentration assayed in duplicate. Growth control wells (bacteria + medium, no peptide) and sterility control wells (medium only) were included. In parallel, the inoculum density and purity were verified by plating serial dilutions of the bacterial culture on MHB agar. After incubation at 37 °C for 20 h under static conditions, bacterial growth was quantified by measuring optical density at 620 nm (OD 620). The MIC was defined as the lowest peptide concentration at which OD 620 showed no increase compared to the growth-control baseline. The experiment was repeated in three independent biological replicates.

#### 3.3.2. Hemolysis Assay

The hemolysis assay was performed using pooled human blood of at least 10 healthy volunteers obtained from healthy donors at the Medical University of Graz (ethical approval: 1142/2025). Erythrocytes were isolated by centrifuging the blood for 10 min at 2100 rpm and subsequently washed several times with PBS. A 2.5% erythrocyte solution in PBS was exposed 10 EB peptides (Figure 2) (final concentrations: 6.25–400 μM). After 1 h at 37 °C under static conditions, the erythrocyte suspensions were centrifuged for 10 min at 4000 rpm and the absorbance of the supernatants was determined at 405 nm. The percentage of hemolysis was calculated relative to the positive control (1% Triton X-100), according to the following equation:

Hemolysis (%) = (Absorbance at peptide × concentration/Absorbance at Triton) × 100

#### 3.3.3. *In Silico* Analysis of Peptides Structural Properties

Peptide structures were predicted using AlphaFold3 Version 4 on a local workstation with Nvidia GeForce RTX 3090 24 GB, AMD Ryzen 9 5900X using 12 cores and 128 GB RAM, which employs a deep learning-based architecture trained on experimentally determined structures to predict atomic-resolution models and provide per-residue confidence metrics (Beta Server, standard settings, 11 November 2024) [25]. Molecular graphics and analyses were performed with UCSF ChimeraX [32]. In addition, peptide sequences were submitted to the PEP-FOLD server (https://bioserv.rpbs.univ-paris-diderot.fr/services/PEP-FOLD/, accessed on 10 December 2025) to generate 3D models using a coarse-grained force field, which were then clustered with Apollo for secondary structure prediction [16,33,34]. The peptide structure are determined with PepFold3 version [21], which uses Markov model sub-optimal conformation sampling approach enabling de novo predictions for peptides comprising 5–50 amino acid in aqueous environment at neutral pH. The basic structural parameters of the peptides, including hydrophobicity, acidity, and charge, were calculated using the Peptide 2.0 hydrophobicity/hydrophilicity analysis tool [https://www.peptide2.com/N_peptide_hydrophobicity_hydrophilicity.php, accessed on 11 November 2025]. Hydrophobic moment (µ), and bilayer partitioning free energy (ΔG) were obtained using Totalizer, a tool within Membrane Protein Explorer (mPEX) [35], [https://blanco.biomol.uci.edu/mpex/, accessed on 11 November 2025]. Totalizer computes peptide hydropathy plots based on whole-residue hydrophobicity according to the Wimley–White scales [36], allowing evaluation of the energetic cost of inserting H-bonded peptide bonds of α-helices into membranes. Bilayer partitioning free energy was assessed using the octanol scale, representing the free energy change for transferring residues from water to the hydrophobic core of the bilayer.

#### 3.3.4. Membrane Permeability Assay

Bacterial membrane permeabilization was evaluated using propidium iodide (PI) uptake. PI is a membrane-impermeable dye: it cannot cross intact bacterial cytoplasmic membranes, but when the membrane integrity is compromised (for example, via peptide-mediated permeabilization), PI enters the cell, intercalates into nucleic acids (DNA and RNA) and fluorescence increases markedly. *B. subtilis*, *E. hirae* and *S. epidermidis* cells were grown to mid-logarithmic phase, harvested by centrifugation, washed once with PBS and diluted to a concentration of 1 × 10^7^ cells/mL using PBS instead of growth medium. The cell suspension was incubated with peptides (Table 1) at various concentrations in the presence of PI (final concentration 2.5 µg/mL). Fluorescence (excitation 535 nm, emission 617 nm) was monitored every 2 min for 120 min at 37 °C using a microplate reader (GloMax^®^, Promega, Madison, WI, USA). The time-course fluorescence curves were recorded for each peptide concentration (in duplicate for each time point, and the entire experiment was repeated three independent times). From each time-course fluorescence curve (for a given peptide concentration), a constant time-window (for example minutes 10 to 20) was selected where the fluorescence signal was stable or in the linear uptake phase. The fluorescence values within that window were averaged to provide a representative fluorescence uptake value for that peptide concentration. These averaged fluorescence values (mean of duplicates) were then plotted as a function of peptide concentration (for each independent experiment) to generate a dose–response curve (fluorescence uptake vs. peptide concentration). The three independent biological replicates were averaged and standard deviations (or standard errors) calculated and plotted.

#### 3.3.5. Membrane Depolarization Assay

The assay was performed as previously described [26] and in analogy to PI assay described above. Briefly, the mid-log phase cells (1 × 10^7^ CFU/mL) were stained with 1 µM DiSC_3_(5) in PBS containing 100 mM KCl for 30 min to allow dye self-assembly and K^+^ equilibration. Peptides of different concentration were added, and fluorescence was recorded at 622/685 nm using was monitored every 2 min for 120 min at 37 °C using a microplate reader (GloMax^®^, Promega, Madison, WI, USA). Further analysis was performed analogously to the PI uptake assay described in Section 3.3.4.

#### 3.3.6. *In Silico* Peptide–Membrane Interactions

Structure of the peptides were predicted by AlphaFold3 Version 4, as described above, with peptides containing free cysteine residues settings. Bacterial membrane models were composed as follow: for *B. subtilis*, 35% 1,2-distearol-sn-glycero-3-phosphoglycerol (DSPG), 35% 1,2-dipalmitoyl-sn-glycero-3-phosphoglycerol (DPPG), 12% phosphatidylethanolamine (PE), 3% cardiolipin (CL), 5% phosphatidylglycerol (PGH), 2% lysyl-mono-galactosyldiacylglycerol (LMG), 4% digalactosyldiacylglycerol (DGD), and 2% bis(monoacylglycero)phosphate (B5S); for *E. hirae*, 30% DSPG, 30% DPPG, and 20% CL; and for *S. epidermidis*, 8% PE, 26% DPPG, 26% DSPG, and 20% CL. These models closely reflect membrane compositions reported in earlier studies [37]. The prediction analysis yielded a ranking score of 0.84, ipTM and pTM scores of 0.34 and 0.35, respectively, and an ipTM + pTM value of about 0.34, and these results were consistent across all models.

### 3.4. Nano-Ultra-Performance Liquid Chromatography-Electrospray Ionization-Quadrupole Mass Spectrometry Analysis

Peptides were desalted on the AssayMAP Bravo Platform (Agilent Technologies, Waldbronn, Germany) using in-house packed mixed-cation exchange (MCX) cartridges. Cartridges were conditioned with methanol (100 μL), equilibrated with ultrapure water (100 μL), and loaded with 100 μL of 0.1 mg/mL peptide solution. Peptides were eluted with 25 μL of 10% ACN in 1% ammonium hydroxide, vacuum-dried, and reconstituted in 0.1% formic acid to a final concentration of 0.1 mg/mL. They were separated on a nanoAcquity UPLC system equipped with a nanoAcquity UPLC 2G-V/M symmetry C18 trap column (100 Å, 5 μm, 180 μm  ×  20 mm) and a nanoAcquity UPLC BEH C18 analytical column (130 Å, 1.7 μm, 100 μm  ×  100 mm) (Waters, Milford, MA, USA). The column temperature was set to 40 °C and injection volume was 1 μL. Mobile phase A consisted of aqueous 0.1% formic acid, and mobile phase B consisted of 0.1% formic acid in 95% ACN. Isocratic delivery of solvent A into the trap column was performed at a flow rate of 15 μL/min for 2 min. The samples were eluted under gradient elution conditions at a flow rate of 1 μL/min and a run time of 32 min. The following elution gradient was used: 0–3 min, 80% solvent A; 3–24 min, 45% solvent A; 24–27 min, 1% solvent A; 27–29 min, 80% solvent A; and 29–32 min, 80% solvent A. The modifier solution of 1 mM ethyl methanoate in isopropanol was introduced from a Synapt A channel by a “T” connector at a flow rate of 0.4 μL/min.

A nanoUPLC system was coupled to a nanoESI-QTOF Synapt G2-Si mass spectrometer (Waters, Milford, MA, USA), and instrument parameters were set using MassLynx software (v4.1, Waters, Milford, MA, USA). Acquisition mode for both MS and MS/MS was set to positive polarity and resolution analyzer mode with the following parameters: nitrogen flow was 1.1 bar with a source temperature of 80 °C, capillary voltage was set to 4.2 kV, cone voltage was set to 40 kV and spectral acquisition time was 1 s. Collision energy during MS/MS was set to 40 V for all peptides. The mass accuracy of the raw data was achieved by infusing 1 ng/μL of leucine enkephalin in isopropanol and 0.1% formic acid. Table A1. contains the list of selected precursor ions.

Data analysis: The obtained MS/MS spectra were deconvoluted using *ExDViewer* software (v4.6.31, Agilent Technologies). The assignment of *y-* and *b*-ion series, as well as sequence coverage calculation, was performed within the same software. MS analysis results are shown in Supplementary Materials: Figures S21–S54 and Tables S2–S11.

## 4. Conclusions

To identify biologically active regions in the Equinin B (EB) sequence, we generated a set of peptides and applied structure–function-driven modifications to enhance their antimicrobial activity. The equinin B amino acid sequence was fragmented at residue 12 (Ser) and 23 (Lys), producing three peptide fragments: **EB1**, **EB2,** and **EB3**. In the first derivatization all Lys residues in **EB1** and **EB2** were replaced with Arg to increase hydrogen bonding (**EB1-K**, **EB2-K**). In the second derivatization all Cys residues were replaced with Arg (**EB1-C**, **EB2-C**, **EB3-C**). All compounds were tested for antimicrobial activity against *E. coli* and *S. epidermidis*. Only peptides from the third part of equinin B, **EB3** (MIC 25 µM), and **EB3-C** (MIC 25–50 µM) showed antimicrobial activity only against the Gram-positive *S. epidermidis*. When Phe was removed from **EB3-C**, the antimicrobial activity was lost in the peptide **EB3a-C**. This removal reduced hydrophobicity from 27% to 20%, suggesting that ~20% represents the minimal threshold for antimicrobial activity, consistent with the inactivity of all peptides below this level. Since **EB3** shows high hemolytic activity, **EB3-C** is the leading peptide for potential drug design. *In silico* structure analysis predicts a better helical structure for **EB3,** while **EB3-C** shows a less folded structure. To verify that the peptides’ effects were not artifacts and not limited to *S. epidermidis*, they were also tested against two additional Gram-positive species—both showing similar potency against *B. subtilis* (MIC ~25–50 µM) and somewhat lower activity against *E. hirae* (MIC ~50–100 µM), suggesting a more general target. *In silico* analysis of their physicochemical properties indicated that **EB3** had the lowest bilayer partitioning free energy (ΔG) and higher amphipathicity, suggesting a greater potential for membrane interaction. **EB3-C** exhibited a slightly higher ΔG and lower amphipathicity, consistent with its predicted reduced membrane affinity, highlighting how these properties may contribute to differences in antimicrobial potency. Evaluation of membrane effects using PI and DiSC_3_(5) assays revealed that **EB3** significantly depolarized the membranes of all tested Gram-positive bacteria, but only markedly increased permeability in *B. subtilis*. In contrast, **EB3-C** induced minimal depolarization and negligible permeabilization, supporting a non-lytic mechanism or one that does not involve lethal disruption of the membrane. Structure–function modeling indicates that **EB3** most likely lacks membrane selectivity due to its ability to adopt multiple non-specific conformations across Gram-positive membranes, including shallow insertion in *B. subtilis*, surface binding in *E. hirae*, and a carpet-like mechanism in *S. epidermidis*. **EB3-C** exhibits consistent, selective interactions via a short amphipathic helix and conserved R4/R6–phosphate contacts, with hydrophobic residues fine-tuning membrane anchoring, which explains its non-hemolytic, targeted antimicrobial activity. In contrast, removal of the phenylalanine residue in **EB3a-C** disrupts proper membrane engagement, leading to primarily surface-bound interactions and loss of antimicrobial function. Among the analyzed peptides, four (**EB1**, **EB2**, **EB1a-K**, and **EB1-K**) showed a propensity to form intramolecular disulfide bonds, whereas **EB3** did not. Regardless of the presence of intra- or intermolecular disulfide interactions, only **EB3** exhibited antibacterial activity. Removal of cysteine from **EB3** did not abolish this activity, indicating that cysteine is not essential for antimicrobial function, whereas removal of phenylalanine resulted in complete loss of activity. Given that **EB3** corresponds to the most hydrophobic region, and supported by structural predictions and experimental data, we conclude that charge, hydrophobicity, and amphipathicity are the primary determinants of antimicrobial activity. These findings demonstrate that **EB3** is the core antimicrobial region of equinin B against Gram-positive bacteria, that substituting C with R preserves antimicrobial activity while reducing hemolysis, and that subtle structural changes can dramatically alter peptide–membrane interactions and biological activity.

## Figures and Tables

**Table 1:** Antimicrobial activity of compounds against *E. coli* and *S. epidermidis*.

Compound	Sequence	*Escherichia coli*	*Staphylococcus epidermidis*
		MIC_90_/µgmL^−1^	MIC_90_/µgmL^−1^
**EB1**	GQCQRKCLGHCS	>100	>100
**EB2**	KKCPKHPQCRK	>100	>100
**EB3**	RCIRRCFGYCL	100	25
**EB1a-K**	GQQRRCLGHCS	>100	>100
**EB-1K**	GQCQRRCLGHCS	>100	>100
**EB-2K**	RRCPRHPQCRR	>100	>100
**EB1-C**	GQRQRKRLGHRS	>100	>100
**EB2-C**	KKRPKHPQRRK	>100	>100
**EB3-C**	RRIRRRFGYRL	>100	25–50
**EB3a-C**	RRIRRRGYRL	>100	>100

**Table 2:** Presentation of the difference in Gibbs energies and the moment of hydration for the prepared peptides, Wimley–White partitioning parameters (amphipathicity = hydrophobic moment, µ; ∆G_W-OCT_, free energy transfer from water to bilayer). The bold was used to emphasize active compounds.

Compound	ΔG	Total Hydrydrophobic Moment
**EB1**	9.93	5.3
**EB2**	16.35	4.48
**EB3**	**1.73**	**6.97**
**EB1a-K**	16.86	5.47
**EB1-K**	16.84	4.42
**EB2-K**	20.29	2.17
**EB1-C**	15.42	4.59
**EB2-C**	20.01	4.01
**EB3-C**	**7.22**	**5.58**
**EB3a-C**	8.93	5.47

## Data Availability

The data for the research results can be obtained from Supporting Information.

## Supplementary Materials

The following supporting information can be downloaded at: https://www.mdpi.com/article/10.3390/md24010046/s1, Table S1: Starting resins with first anchored amino acids and final peptides obtained with SPPS; Figures S1–S20: NMR spectra of prepared peptide EB derivatives; Figures S21–S50: MS analysis of peptide EB derivatives. Tables S2–S11: Summary of MS/MS identification metrics for peptides obtained by ExDViewer software; Figures S51–S54: MS analysis of peptides for disulfide bonds detection.

## Abbreviations

The following abbreviations are used in this manuscript:

AR	Antibiotic resistance
AMPs	Antimicrobial peptides
HBTU	*N*,*N*,*N′*,*N′*-Tetramethyl-*O-*(1*H*-benzotriazol-1-yl)uronium hexafluorophosphate
NMM	*N*-methylmorpholine
TFA	Trifluoroacetic acid
TIS	Triisopropylsylane
SPE	Solid Phase Extraction
SPPS	Solid Phase Peptide Synthesis
MIC	Minimum Inhibitory Concentration
PI	Propidium iodide
MHB	Mueller–Hinton Broth
DMSO	Dimethyl sulfoxide
DMF	Dimethylformamide

## Appendix A

**Table A1:** List of selected precursor ions and their respective retention times (Rt) used in MS/MS analysis.

Peptide ID		Peptide Sequence	Precursor *m*/*z*	Rt/min
1	**EB1**	GQCQRKCLGHCS	[660.2925 + 2H]^2+^	11.53
2	**EB-2**	KKCPKHPQCRK	[676.8743 + 2H]^2+^	09.17
3	**EB3**	RCIRRCFGYCL	[695.3392 + 2H]^2+^	18.06
4	**EB1a-K**	GQQRRCLGHCS	[622.7909 + 2H]^2+^	10.17
5	**EB1-K**	GQCQRRCLGHCS	[674.2955 + 2H]^2+^	10.77
6	**EB2-K**	RRCPRHPQCRR	[732.8866 + 2H]^2+^	09.17
7	**EB1-C**	GQRQRKRLGHRS	[739.9304 + 2H]^2+^	09.76
8	**EB2-K**	KKRPKHPQRRK	[729.9662 + 2H]^2+^	08.76
9	**EB3-C**	RRIRRRFGYRL	[774.9771 + 2H]^2+^	09.07
10	**EB3a-C**	RRIRRRGYRL	[701.4429 + 2H]^2+^	09.23
