# Supplementary material for: Truncated Equinin B Variants Reveal the Sequence Determinants of Antimicrobial Selectivity
Source: Mar Drugs. 2026 Jan 17;24(1):46. doi: 10.3390/md24010046 (PMC12842892; doi:10.3390/md24010046)
Supplement: Supplementary file 1 [file marinedrugs-24-00046-s001.zip › marinedrugs-4040894-supplementary.pdf]

Supplementary for:

Truncated Equinin B variants reveal the sequence determinats of antimicrobial selectivity

Mariele Staropoli,[1,2,#] Theresa Schwaiger,[3,#] Jasmina Tuzlak,[3,#] Renata Biba,[4] Lukas Petrowitsch,[3] Johannes Fessler,[5] Marin Roje,[1] Matteo Cammarata,[2] Nermina Malanović,[3,6,7,*] Andreja Jakas[1,*]

[1] Laboratory for Chiral Technologies, Division of Organic Chemistry and Biochemistry, Ruđer Bošković Institute, Bijenička c. 54, 10000 Zagreb, Croatia
[2] Department of Earth and Marine Sciences, University of Palermo, , Viale delle Scienze, Ed. 16, Palermo, 90128 Italy
[3] Institute of Molecular Bioscience, University of Graz, Humboldtstr.50, 8010 Graz, Austria
[4] Division of Molecular Medicine, Ruđer Bošković Institute, , Bijenička c. 54, 10000 Zagreb, Croatia
[5] Division of Immunology, Otto Loewi Research Center, Medical University of Graz, , Graz, Austria
[6] Field of Excellence Biohealth
[7] BioTechMed Graz
[#] These authors participate equally.
* Correspondence: andreja.jakas@irb.hr and nermina.malanovic@uni-graz.at

Table of contents

1. **Peptide synthesis strategy** **S2**
2. **NMR spectra of prepared peptides EB derivatives** **S4**
3. **MS analysis of peptides EB derivatives** **S19**
4. **MS analysis of peptides for disulfide bond detection** **S30**

1. Peptide synthesis strategy

Table S1. Starting resins with first anchored amino acids and final peptides obtained with SPPS.

Starting resin for SPPS	Final peptide*
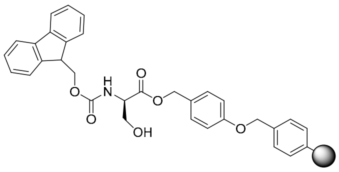 Fmoc-Ser-Wang resin	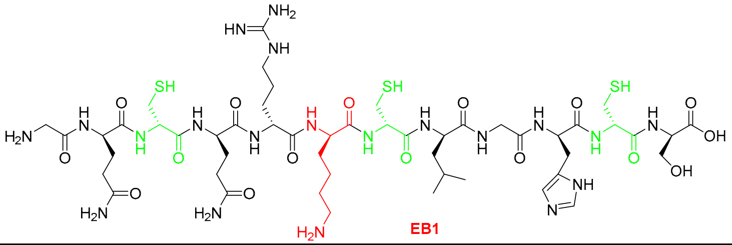 EB1
	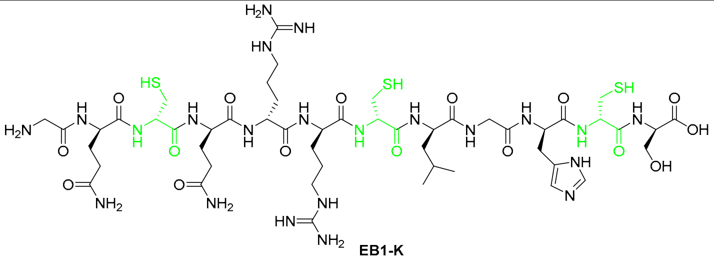 EB1-K
	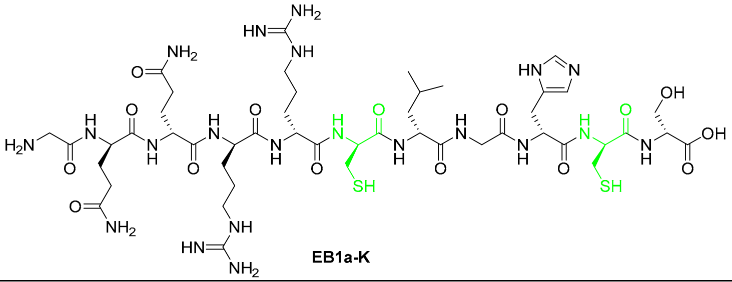 EB1a-K
	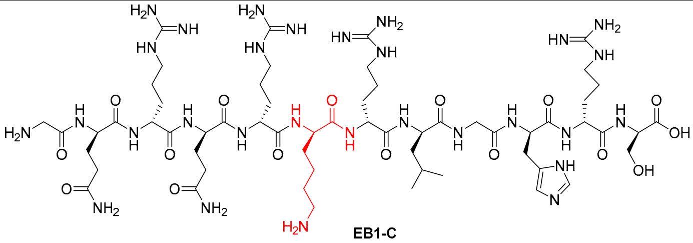 EB1-C
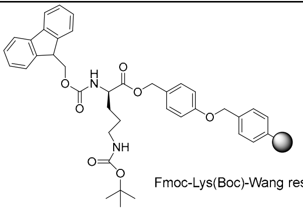 Fmoc-Lys(Boc)-Wang resin	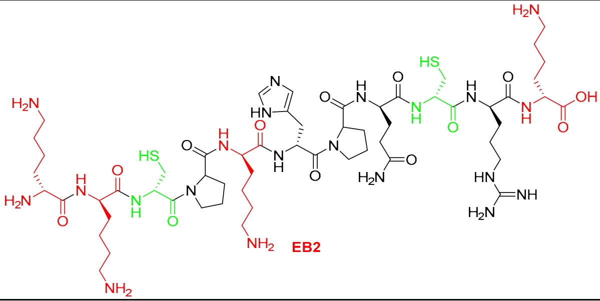 EB2

	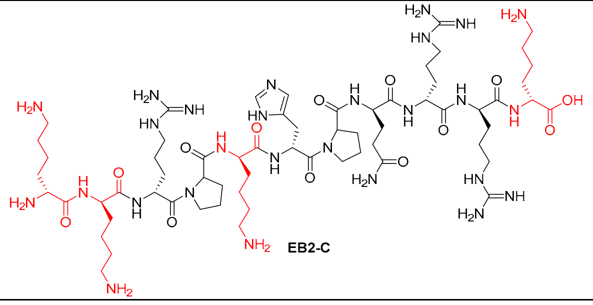 EB2-C
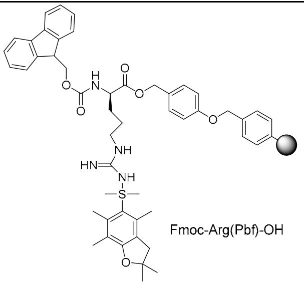 Fmoc-Arg(Pbf)-OH	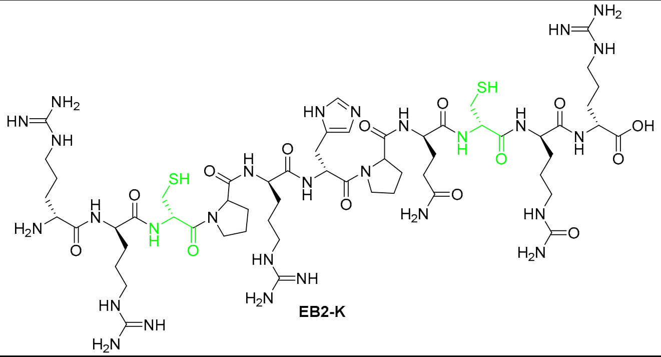 EB2-K
	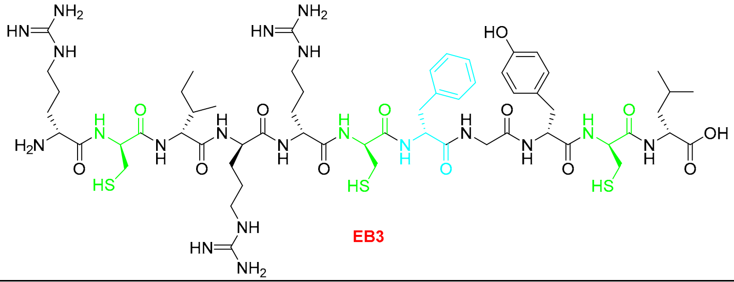 EB3
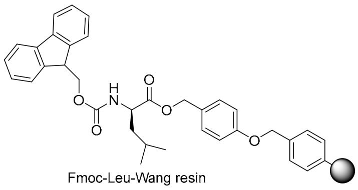 Fmoc-Leu-Wang resin	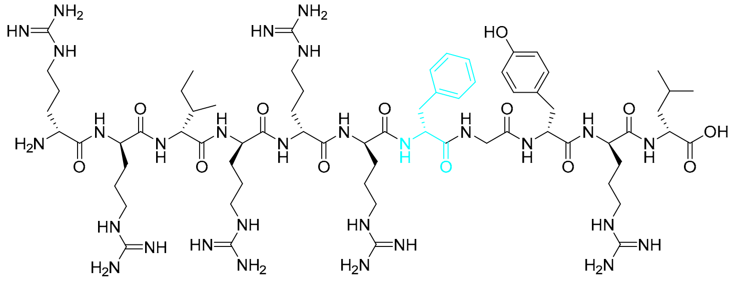 EB3-C
	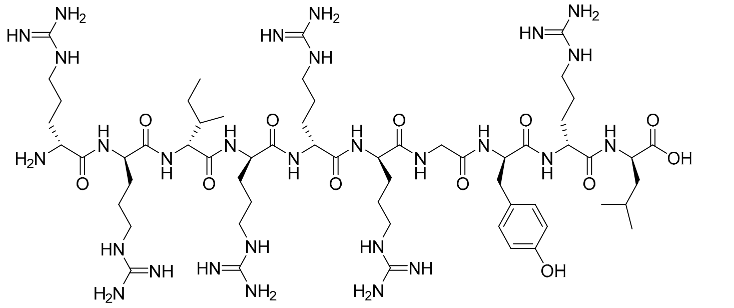 EB3a-C

*The amino acids in the truncated peptides where changes were made to the original equinin B sequence are colored differently from the other amino acids. In the derivatives, the new amino acids

(Arg) are shown in black. The changes are made to Lys (red) and Cys (green), which are substituted with Arg, or some amino acids are simply removed (Cys in **EB1a-K**, Phe (blue) in **EB3a-C**).

2. NMR spectra of prepared peptide EB derivatives

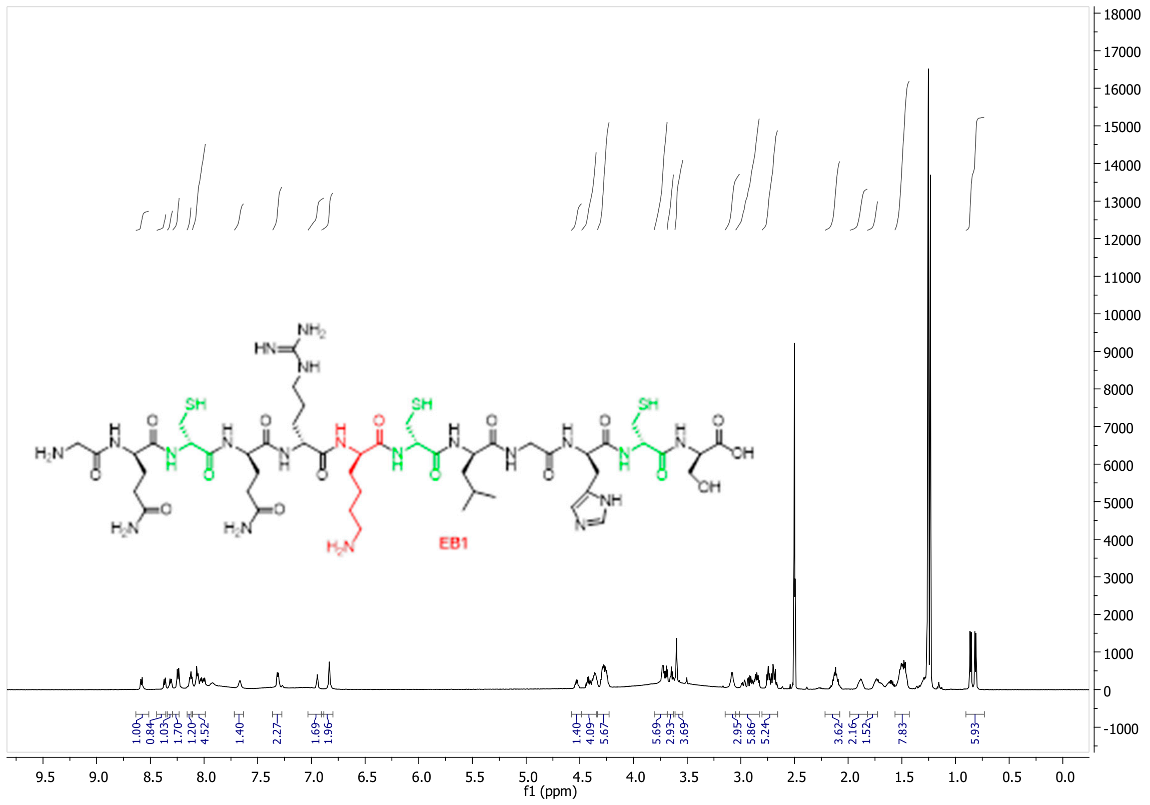

Figure S1. ^{1}H NMR spectrum of peptide **EB-1** in DMSO-d_6

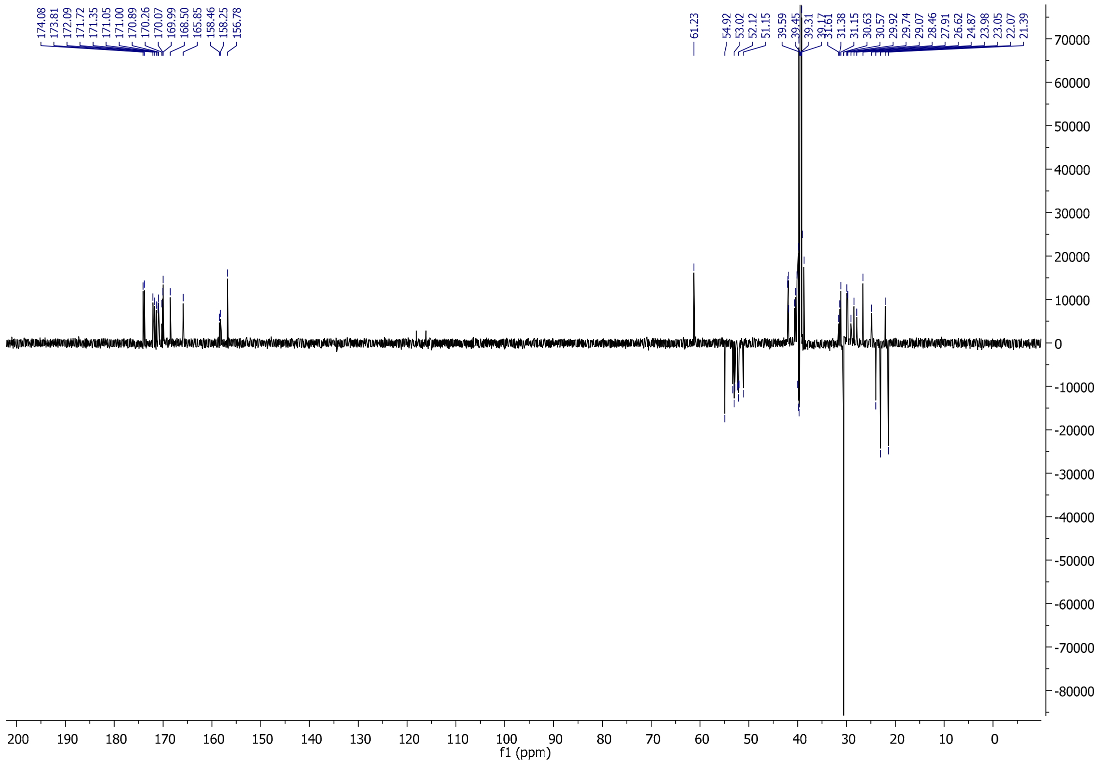

Figure S2. ^{13}H NMR spectrum of peptide **EB1** in DMSO-d_6

EB1 ^{1}H NMR (600 MHz, DMSO) δ 8.58 - 7.31 NH, 6.94 (s, 0.3H) NH-His, 6.83 (s, 0.7H) NH-His, 4.53 (dd, *J* = 12.8, 7.5 Hz, 1H) α-Gln, 4.42 (dt, *J* = 9.8, 6.4 Hz, 1H) α-Gln, 4.39 – 4.33 (m), 4.31 – 4.22 (m, 3H) α-His, α-Ser, α-Leu, 3.73 (d, *J* = 6.2 Hz, H) α-Gly, 3.70 (dd, *J* = 11.0, 5.4 Hz, 1H) β-Ser, 3.64 (dd, *J* = 11.0, 4.5 Hz, 1H) β-Ser, 3.60 (s, 2H) δ-Arg, 3.08 (d, *J* = 5.9 Hz, 2H) δ-Arg, 3.02 – 2.96 (m) β-His, 2.94 – 2.89 (m) β-Ser, 2.89 – 2.82 (m) β-His, 2.78 – 2.73 (m) ε-Lys, 2.71 – 2.66 (m) ε-Lys, 2.18 – 2.06 (m, 4H) γ-Gln, 1.95 – 1.85 (m, 2H) β-Cys, 1.79 – 1.68 (m, 4H) β-Cys, 1.60 (dd, *J* = 13.6, 6.9 Hz, 2H) γ-Leu, 1.56 – 1.42 (m, 8H) β-Lys, β-Leu, β-Cys, 1.37 – 1.28 (m, 2H) γ-Lys, 0.86 (d, *J* = 6.6 Hz, 3H) δ-Leu, 0.81 (d, *J* = 6.5 Hz, 3H) δ’-Leu. ^{13}C NMR (151 MHz, DMSO) δ 174.08 - 165.85 CO, 156.8 ε-Arg, 134.4 ε-His, 132.5 γ-His, 118.10 δ-His, 61.23 Ser-β, 54.9 α-His, 53.3 α-Ser, 53.0 α-Gln, 52.8 α-Cys, 52.3 α-Gln, 52.2 α-Cys, 52.1 α-Cys, 52.1 α-Lys, 52.0 α-Arg, 51.2 α-Leu, 42.0 α-Gly, 41.9 α-Gly, 41.87, 40.6 β-Leu, 40.4 δ-Arg, 40.1 δ-Arg, 40.0 - 39.0 DMSO, 38.7 ε-Lys, 31.6 β-His, 31.4 γ-Gln, 31.2 γ-Gln, 30.63, 30.6 *t*-Bu-OH, 29.9 β-Arg, 29.7 β-Arg, 29.1 β-Cys, 28.5 β-Cys, 27.9 β-Cys, 26.6 β-Lys, 24.9 γ-Arg, 24.0 γ-Leu, 23.1 Leu-δ, 22.1 γ-Lys, 21.4 Leu-δ’

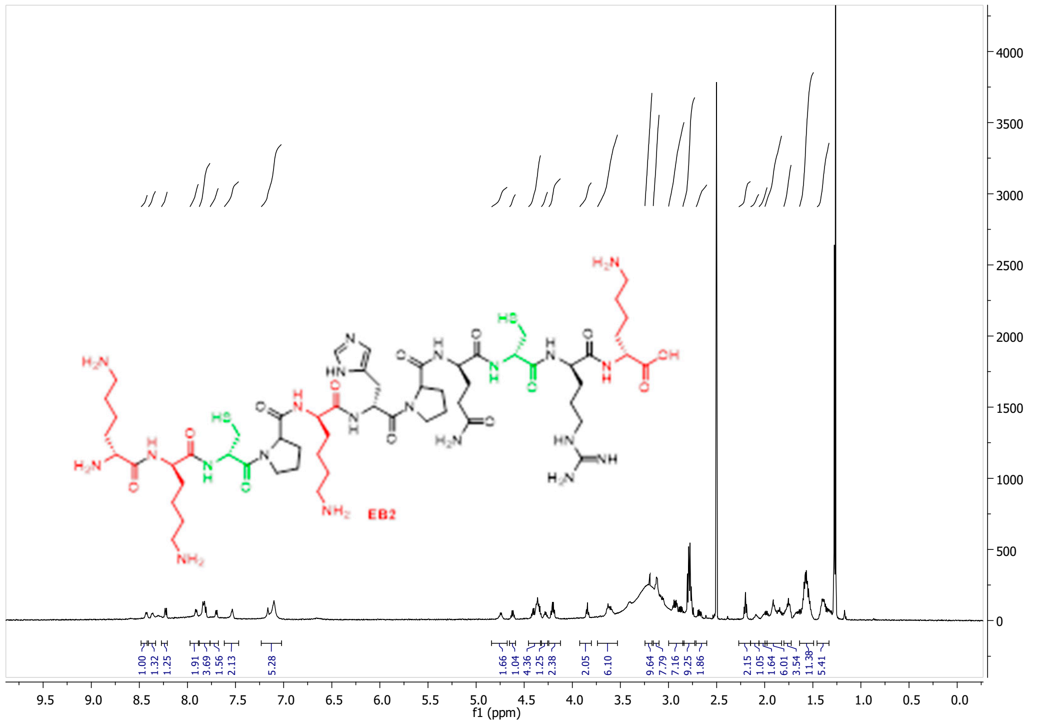

Figure S3. ^{1}H NMR spectrum of peptide **EB2** in DMSO-d_6

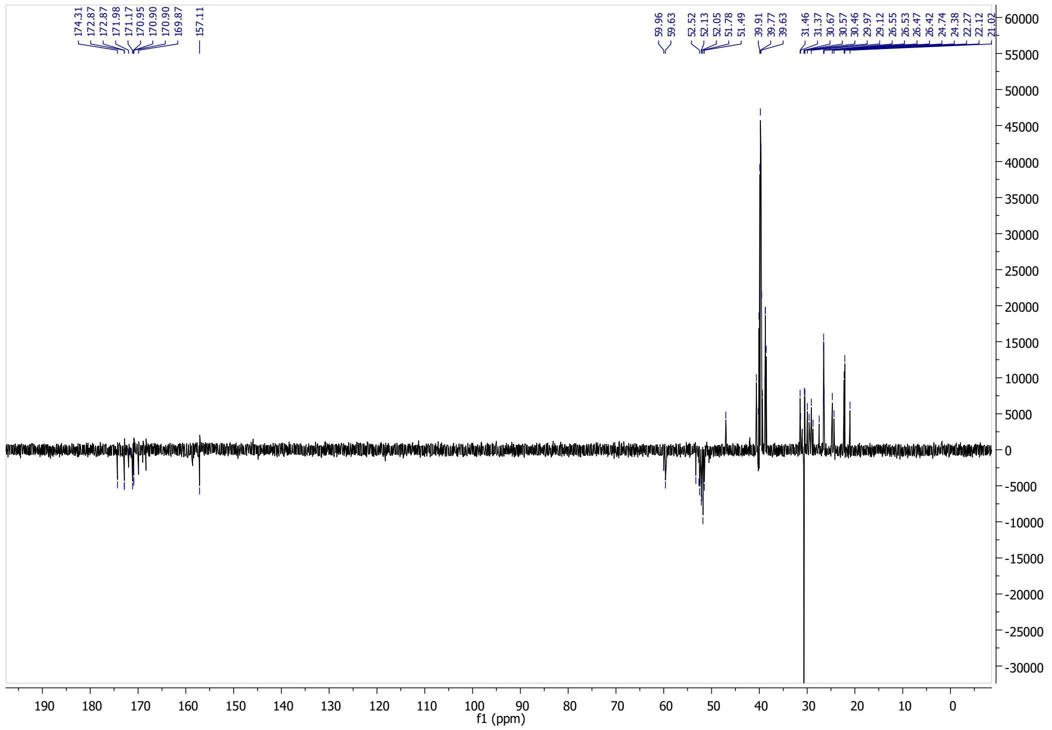

Figure S4. ^{13}H NMR spectrum of peptide **EB2** in DMSO-d_6

EB2 ^{1}H NMR (600 MHz, DMSO) δ 8.42 - 7.10 NH, 4.75 (s, 1H), 4.62 (d, *J* = 7.1 Hz, 1H), 4.46 – 4.32 (m, 4H) α-Pro, α-Lys 4.28 (m, 1H) α-Gln, 4.20 (dd, *J* = 13.5, 8.2 Hz, 2H) α-Lys, 3.84 (t, *J* = 6.5 Hz, 2H) α-Lys, 3.61 (m, 2H) δ-Pro, 3.40 (m) δ-Pro, 3.15 – 3.03 (m) δ-Arg, 2.93 (dd, *J* = 12.9, 5.9 Hz, 2H) β-Pro, 2.89 – 2.84 (m) β-Pro, 2.77 (m) β-Pro, ε-Lys, 2.68 (dd, *J* = 13.0, 7.3 Hz, 1H) β-Pro, 2.20 (t, *J* = 7.6 Hz, 2H) γ-Gln, 2.09 (m, 1H) β-Gln, 1.99 (m) β-Cys, 1.91 (m) β-Gln, 1.84 (m, 2H) β-Cys, 1.76 (m, 4H) δ-Lys, 1.64 (m, 2H) δ-Lys, 1.63 – 1.49 (m) β-Lys, 1.46 – 1.31 (m) δ-Lys, γ-Lys. ^{13}C NMR (151 MHz, DMSO) δ 174.3 - 169.9 CO, 157.11 ε-Arg, 59.9 α-Pro, 59.6 α-Pro, 53.3 α-His 52.6 α-Arg, 52.5 α-Gln, 52.4, 52.13 α-Cys, α-Cys, 52.1 α-Lys, 51.8 α-Lys, 51.5 Lys, 50.5 α-Lys, 47.00 δ-Pro, 40.6 δ-Arg, 40.0 - 39.4 DMSO, 38.8 ε-Lys, 38.7 ε-Lys, 38.6 ε-Lys, 31.5 γ-Gln, 31.4 β-Gln, 31.0 β-His, 30.7 *t*-Bu-OH, 30.6 -δ-Lys, 30.5 δ-Lys, 30.0 δ-, 29.6 β-Pro 29.1 β-Pro, 27.5 β-Cys, 26.6 β-Lys, 26.5 β-Lys, 26.5 β-Lys, 26.4 β-Lys, 24.7 γ-Arg, 24.4 γ-Pro, 24.3 γ-Pro 22.3 γ-Lys, 22.1 γ-Lys, 21.0 γ-Lys.

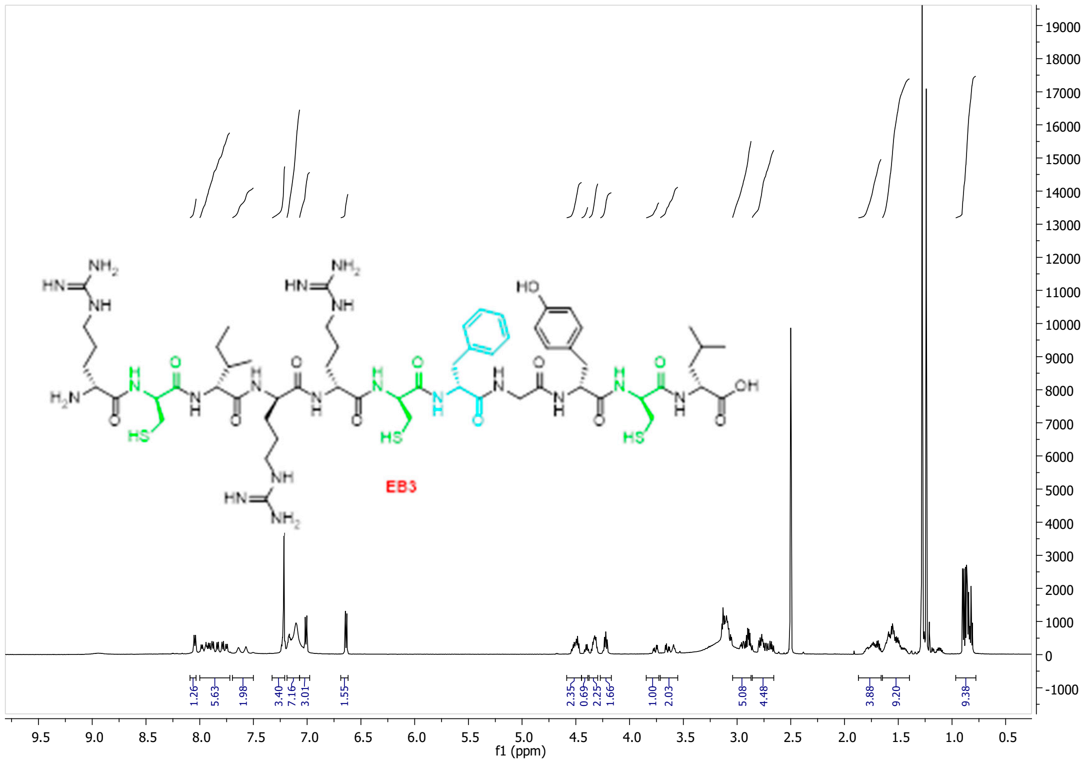

Figure S5. ^{1}H NMR spectrum of peptide **EB3** in DMSO-d_6

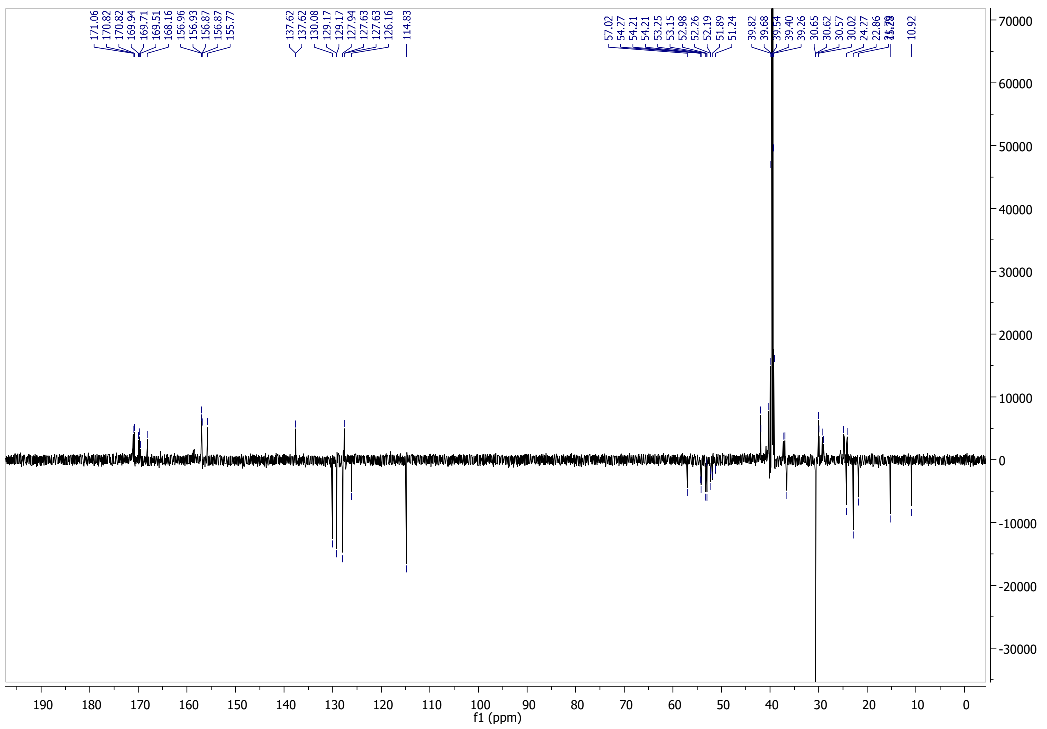

Figure S6. ^{13}H NMR spectrum of peptide **EB3** in DMSO-d_6

EB3 ^{1}H NMR (600 MHz, DMSO) δ 8.94 NH-Leu, 8.05-7.50 NH, (8.16 d, NH-Arg, NH-Cys; 7.99 d, NH-Arg; 7.94 dd, NH-Gly; 7.92 d, NH-Tyr; 7.89 NH-Ile; 7.84 d, NH-Phe; 7.79 d, NH-Cys; 7.74 d, NH-Leu; 7.24- 7.17 (m) δ-Phe, ε-Phe, ξ-Phe, 7.16- 7.10 NH, 7.02-7.00 (d, *J* = 8.4 Hz) δ-Tyr; 6.65-6.63 (d, *J* = 8.4 Hz) ε-Tyr, 4.54-4.47 (m) α-Cys, α-Arg, 4.42-4.38 (m) α-Arg, 4.36-4.30 (m) α-Tyr, α-Phe, 4.25-4.21 (m) α-Ile, α-Leu, 3.78-3.71 (m) α-Gly, 3.67-3.62 (m) α-Gly, 3.59-3.58 α-Arg, 3.14-3.05 δ-Arg, β-Phe, 2.97-2.86 (m) β-Tyr, β-Phe, β-Cys, β-Arg, 2.80-2.66 (m) β-Arg, β-Cys, β-Tyr, β-Ile, 1.81-1.65 (m) β-Ile, 1.62-1.41 (m) γ-Ile, γ-Leu, γ-Arg, γ-Ile, 1.19 -1.08 (m) γ-Ile, 0.95-0.81 (m) δ-Leu, δ-Ile, CH_3-(β-Ile). ^{13}C NMR (151 MHz, DMSO) δ 171.1 CO-Phe, Tyr, 170.8 CO- Ile, Arg, 170.8 CO-Arg, 169.9, 169.7 CO-Cys, 169.5 CO-Leu, 168.2 CO-Gly, 157.0 ε-Arg, 156.9 ε-Arg, 156.9 ε-Arg, 155.8 ξ-Tyr, 137.6 γ-Phe, 130.1 δ-Phe, 129.2 ε-Phe, 127.9 δ-Tyr, 127.6 γ-Ty, 126.2, 114.8 ε-Tyr, 57.0 α-Ile, 54.3 α-Cys, 54.2 α-Phe, 53,3 α-Arg, 53.2 α-Tyr, 53.0 α-Arg, 52.3 α-Cys, 52.2 α-Cys, 51.9 α-Arg, 51.2 α-Leu, 42.0 β-Gly; 40.3 δ-Arg; 37.3 β-Phe, 37.0 β-Tyr, 36.5 β-Ile, 30.0 β-Cys, 29.9 β-Cys, 29.3 β-Arg, 29.0 β-Arg, 24.9 γ-Arg, 24.3 γ-Arg, 24.1 γ-Leu, 22.9 δ-Leu, 21.8 δ-Leu, 15.3 CH_3-(β-Ile), 10.9 δ-Ile.

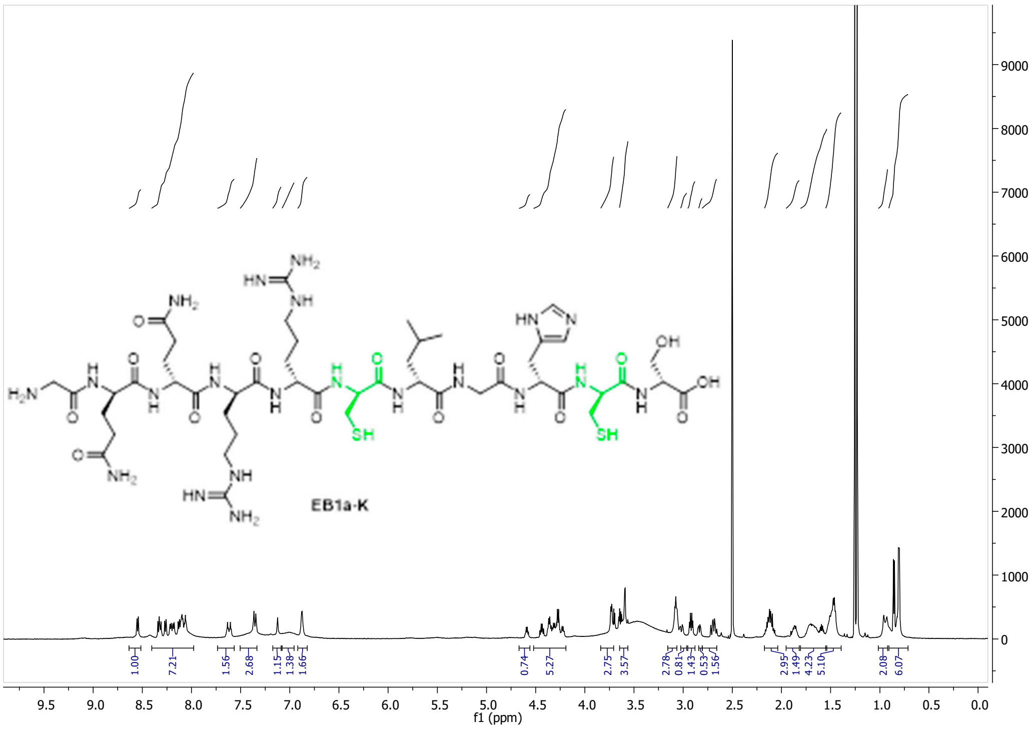

Figure S7. ^{1}H NMR spectrum of **EB1a-K** in DMSO-d_6

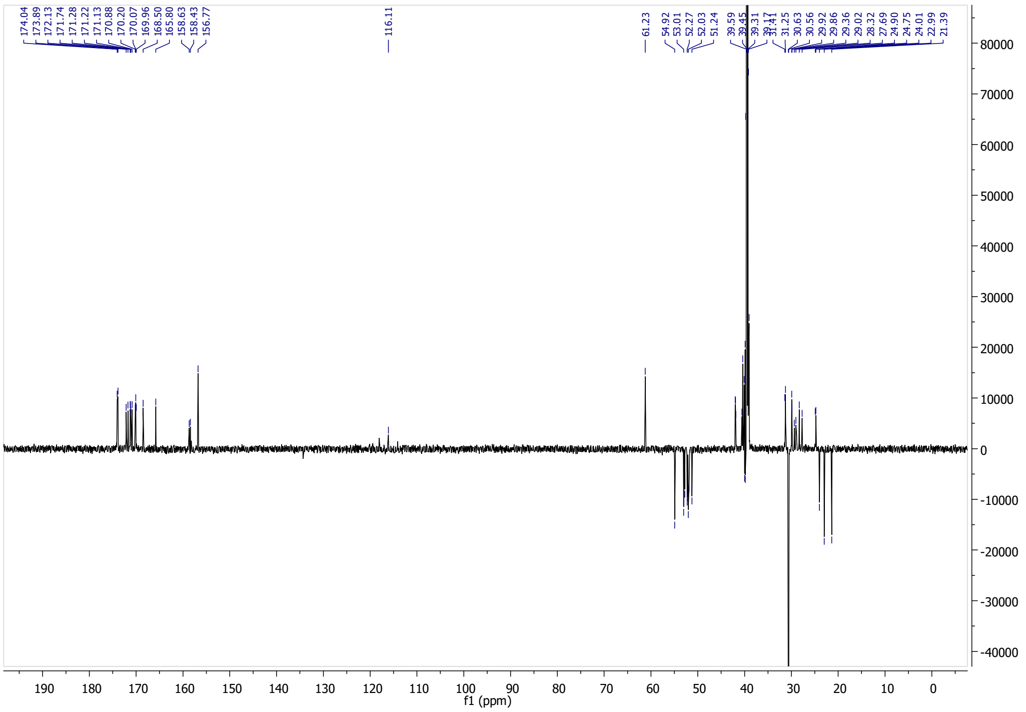

Figure S8. ^{13}H NMR spectrum of **EB1a-K** in DMSO-d_6

EB1a-K ^{1}H NMR (600 MHz, DMSO) δ 8.55 - 6.88 NH, 4.59 (dd, *J* = 13.2, 7.6 Hz, 1H) α-Arg, 4.44 (td, *J* = 8.4, 5.2 Hz, 1H), 4.36 (dt, *J* = 13.8, 6.9 Hz, 2H), 4.34 – 4.29 (m, 2H) α-Gln, 4.29 – 4.25 (m, 2H) α-His, 4.23 (dd, *J* = 14.0, 7.9 Hz, 1H) α-Gln, 3.75 – 3.69 (m, 4H) β-Ser, α-Gly, 3.66 – 3.62 (m, 2H) β-Ser, α-Gly 3.59 (d, *J* = 2.8 Hz, 2H) δ-Arg, 3.11 – 3.05 (m, 5H) δ-Arg, 3.04 – 2.99 (m, 2H) β-His, 2.92 (dd, *J* = 12.9, 5.1 Hz, 2H) β-His, 2.84 (dd, *J* = 12.6, 6.1 Hz, 1H) β-Cys, 2.69 (td, *J* = 13.3, 8.5 Hz, 2H), 2.19 – 2.05 (m, 4H) γ-Gln, 1.93 – 1.82 (m, 2H) β-Gln, 1.70 (d, *J* = 5.4 Hz, 5H) β-Gln, 1.63 – 1.56 (m, 2H) γ-Leu, β-Arg , 1.47 (m, 8H) β-Leu, β-Arg, γ-Arg, 0.94 (d, *J* = 18.4 Hz, 4H), 0.86 (d, *J* = 6.6 Hz, 3H) δ-Leu, 0.81 (d, *J* = 6.5 Hz, 3H) δ'-Leu. ^{13}C NMR (151 MHz, DMSO) δ 174.0 - 165.8 NH, 156.8 ε-Arg, 134.6 ε-His, 132.3 γ-His, 116.1 δ-His, 61.2 β-Ser, 54.9 α-His, 53.0 α-Cys, 52.8 α-Ser, 52.3 α-Gln, 52.2 α-Arg, 52.0 α-Arg, 51.9 α-Arg, 51.2 α-Leu, 42.0 α-Gly, 41.9 α-Gly, 40.6 β-Leu, 40.4 δ-Arg, 40.1 δ-Arg, 40.0 - 39.0 DMSO, 31.4 γ-Gln, 31.3 γ-Gln, 30.6 *t*-Bu-OH, 29.9 β-Arg, 29.9 β-His, 29.4 β-Arg, 29.0 β-Gln, 28.3 β-Arg, 27.7 β-Cys, 24.9 γ-Arg, 24.8 γ-Arg, 24.0 γ-Leu, 23.0 δ-Leu, 21.4 δ'-Leu.

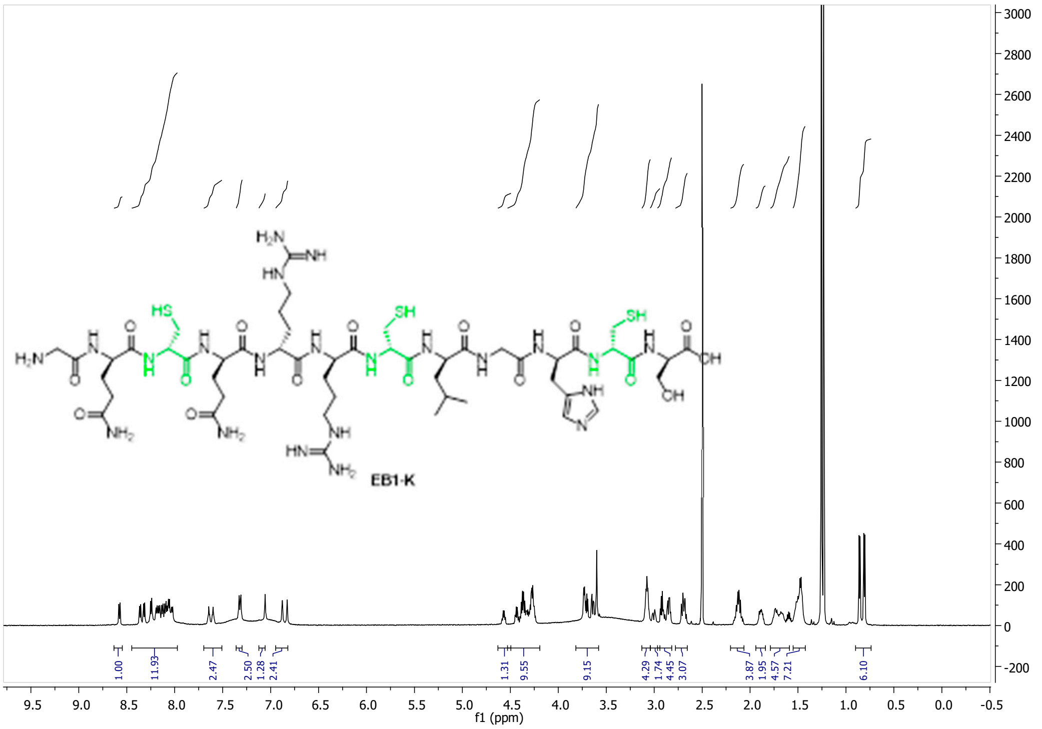

Figure S9. ^{1}H NMR spectrum of **EB1-K** in DMSO-d_6

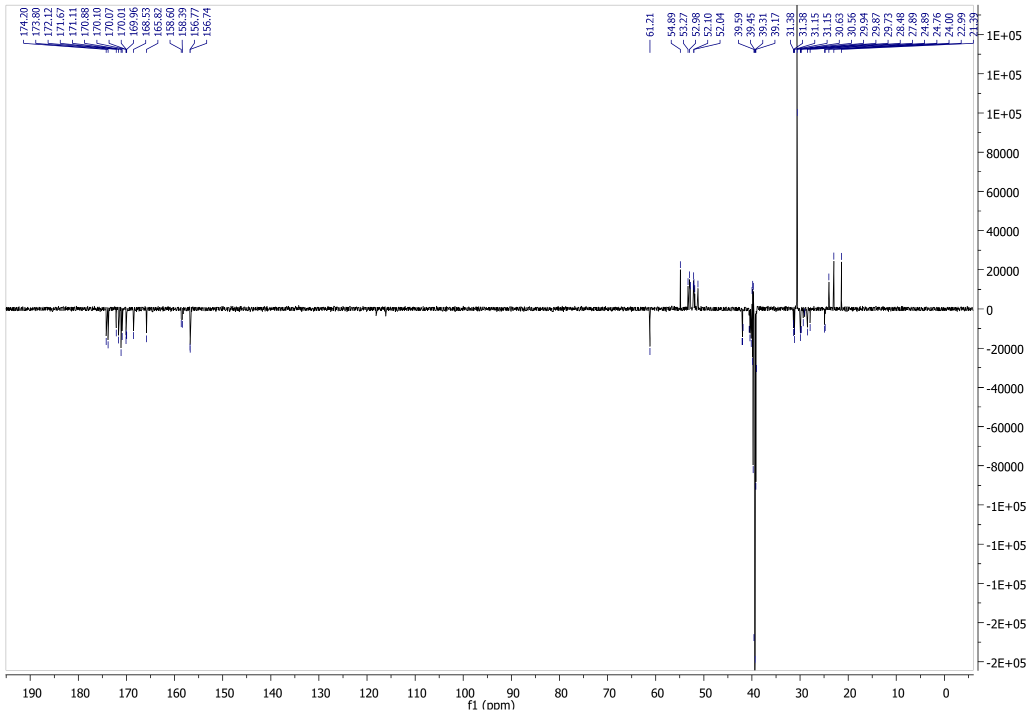

Figure S10. ^{13}H NMR spectrum of peptide **EB1-K** in DMSO-d_6

EB1-K ^{1}H NMR (600 MHz, DMSO) δ 8.58 - 6.83 NH, 4.59 - 4.55 (m) α-Arg, 4.45 - 4.41 (m) α-Arg, 4.40 - 4.31 (m) α-Gln, 4.29 - 4.24 (m) α-Leu, α-His, α-Ser, 3.74 - 3.73 (m) α-Gly, 3.72 - 3.69 (m) β-Ser, 3.65 - 3.63 (m) β-Ser, 3.60 δ-Arg, 3.09 - 3.07 (m) δ-Arg, 3.02 - 2.89 (m) β-His, 2.87 - 2.70 (m) δ-Arg, 2.68 - 2.61 (m) β-His, 2.52 - 2.38 DMSO, 2.14 - 2.08 (m) γ-Gln, 1.91 - 1.88 (m) β-Gln, 1.74 - 1.72 (m) β-Gln, 1.68 – 1.64 (m) β-Arg, 1.62 - 1.58 (m) γ-Leu, 1.50 - 1.47 (m) β-Leu, 1.36 - 1.12 *t*-Bu-OH, 0.86 (d, J = 6.6 Hz, 1H) δ-Leu, 0.81 (d, J = 6.5 Hz, 1H) δ'-Leu. ^{13}C NMR (151 MHz, DMSO) δ 174.2 δ-Gln, 173.8 δ-Gln, 172.1 CO-Leu, 171.7 CO-Gln, 171.1 CO-Gln, 170.9 CO-Arg, 170.1 CO-Arg, 170.1 CO-His, 170.0 CO-Gly, 170.0 CO-Ser, 168.5 CO-Cys, 165.8 CO-Cys, 156.7 ε-Arg, 156.7 ε-Arg, 61.2 β-Ser, 54.9 α-His, 53.3 α-Cys, 53.0 α-Ser, 52.8 α-Arg, 52.2 α-Cys, 52.1, 52.04, 51.8 α-Arg, 51.2 α-Gln, 42.1 α-Gly, 41.9 α-Gly, 41.9, 41.85, 40.5 β-Leu, 40.4 δ-Arg, 40.1 δ-Arg, 40.0 - 39.0 DMSO, 31.4 γ-Gln, 31.2 γ-Gln, 30.6 β-His, 30.6 β-Arg, 29.9 β-Arg, 29.9 β-Cys, 29.7 β-Cys, 29.3 β-Cys, 28.5 β-Gln, 27.9 β-Gln, 24.9 γ-Arg, 24.8 γ-Arg, 24.0 γ-Leu, 23.0 δ-Leu, 21.4 δ'-Leu.

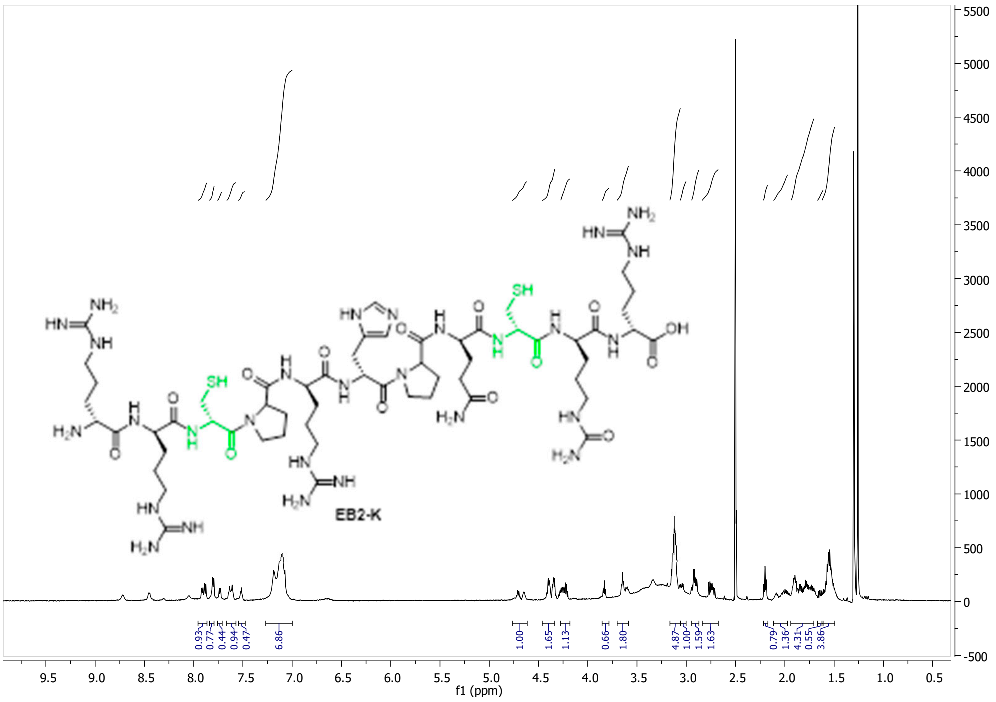

Figure S11. ^{1}H NMR spectrum of peptide **EB2-K** in DMSO-d_6

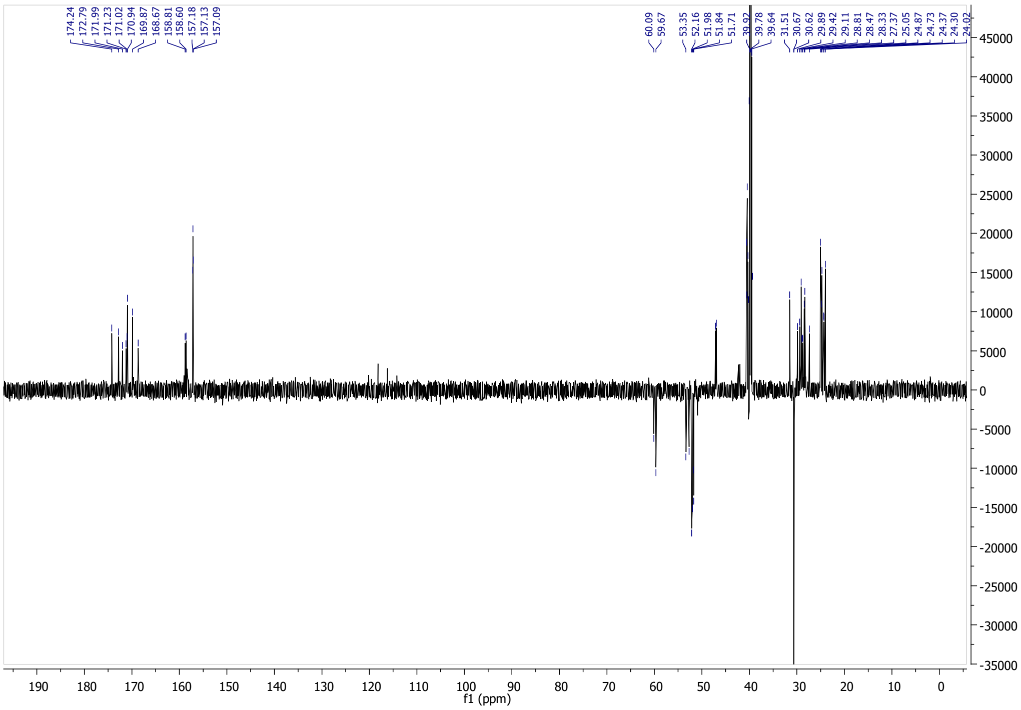

Figure S12. ^{13}H NMR spectrum of peptide **EB2-K** in DMSO-d_6

EB2-K ^{1}H NMR (600 MHz, DMSO) δ 8.72 - 7.12 NH, 4.70 (dd, J = 13.7, 6.6 Hz, 1H) α-Arg, 4.67 – 4.62 (m, 1H) α-Arg, 4.40 (dd, J = 11.7, 6.1 Hz, 2H) α-Pro, α-Cys, 4.37 – 4.32 (m, 2H) α-Pro, α-Cys, 4.31 – 4.20 (m, 3H) α-

Arg, 3.83 (t, J = 6.3 Hz, 1H) α-Arg, 3.64 (m) δ-Pro, 3.68 -3.58 (m) δ-Pro, 3.37 – 3.31 (m) δ-Pro, 3.17 – 3.10 (m) δ-Arg, 3.08 -3.02 (m) β-Pro, 2.95 -2.89 (m) β-Pro, 2.77 – 2.71 (m) β-Pro, 2.20 (t, J = 7.6 Hz, 2H) γ-Gln, 2.12 -2.06 (m) β-Arg, 2.04 – 1.95 (m) β-Cys, 1.93 -1.89 (m) β-Arg, 1.88 – 1.82 (m) β-Cys, 1.80 – 1.75 (m) β-His, 1.76 -1.70 β-His, β-Arg, 1.67 – 1.61 (m) β-Arg, 1.59 – 1.48 (m) γ-Arg. ^{13}C NMR (151 MHz, DMSO) δ 174.4 - 168.7 CO, 157.2 ε-Arg, 157.1 ε-Arg, 157.1 ε Arg, 116.3 δ-His, 60.1 α-Pro, 59.7 α-Pro α, 53.4 α-Arg, 52.7 α-His, 52.2 α-Cys, 52.0 α-Arg, 51.8 α-Arg, 51.7 α-Arg, 47.1 δ-Pro, 46.9 δ-Pro δ 40.6 δ-Arg, 40.5 δ-Arg, 40.5 δ-Arg, 40.3 δ-Arg, 40.2 δ-Arg, 40.0 - 39.4 DMSO, 31.5 γ-Gln 30.6 *t*-Bu-OH, 29.9 β-Pro, 29.4 β-Pro, 29.1 β-Arg, 28.9 β-Cys, 28.8 β-Arg, 28.5 β-Arg, 28.3 β-His, 27.4 β-Cys, 25.1 γ-Arg, 24.9 γ-Arg, 24.7 γ-Arg, 24.4 γ-Pro, 24.3 γ-Pro, 24.0 γ-Arg.

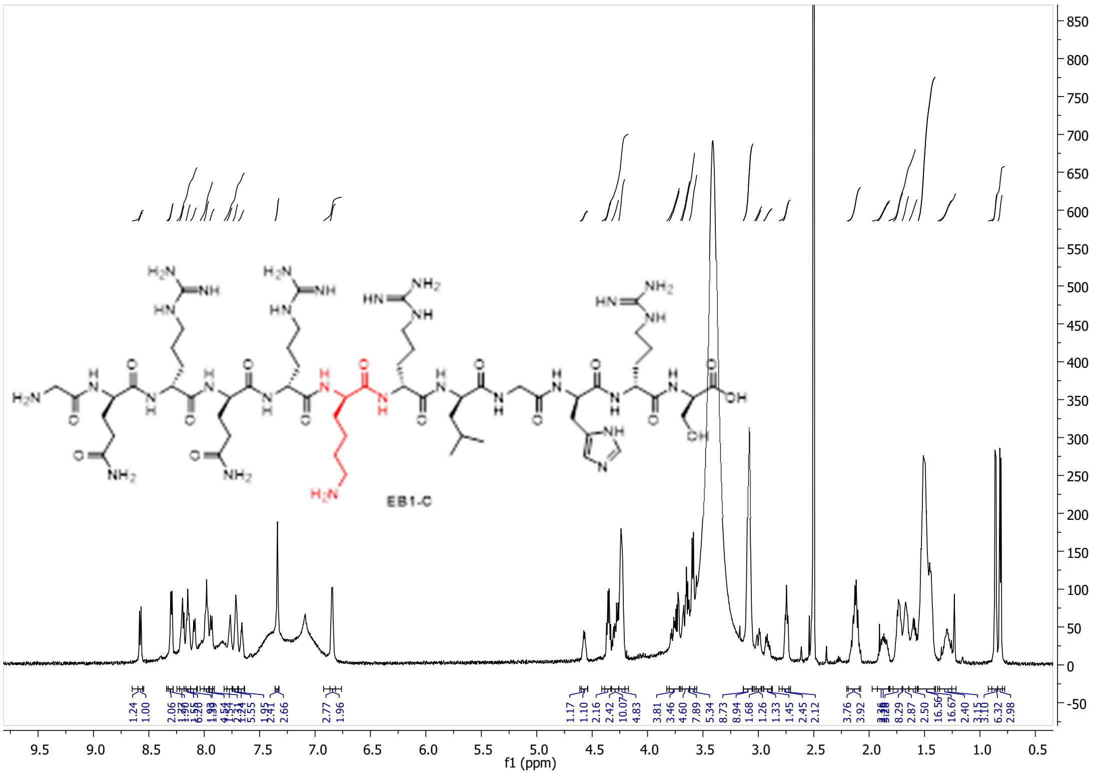

Figure S13. ^{1}H NMR spectrum of peptide **EB1-C** in DMSO-d_6

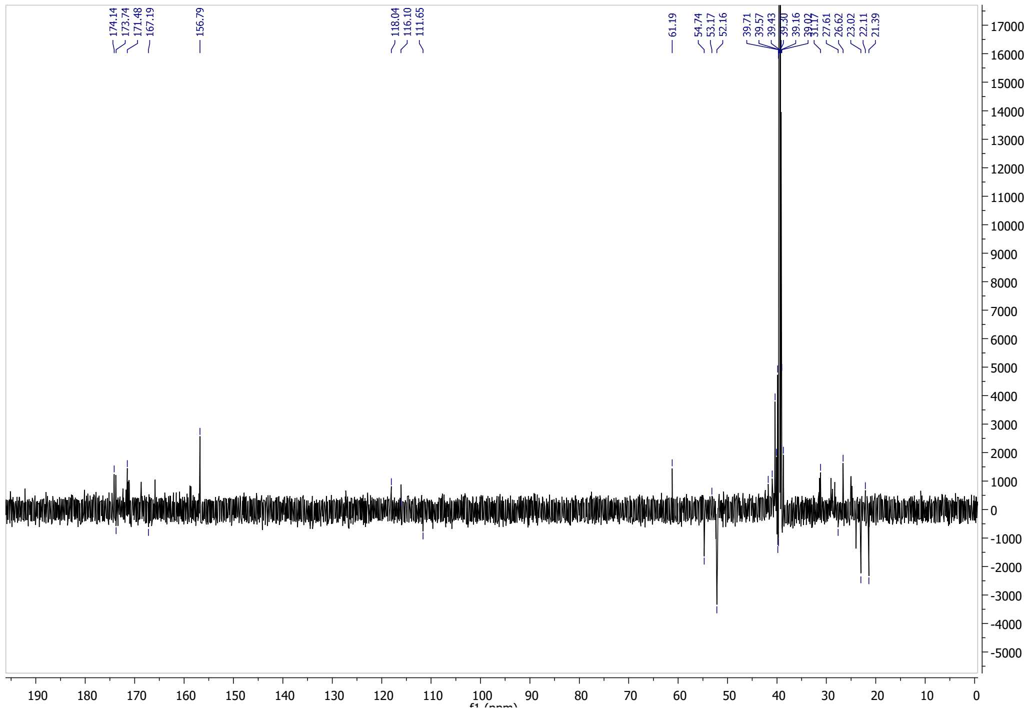

Figure S14. ^{13}H NMR spectrum of peptide **EB1-C** in DMSO-d_6

EB1-C ^{1}H NMR (600 MHz, DMSO) δ 8.58 - 6.85 NH, 4.59 – 4.55 (m, 1H) α-Arg, 4.35 (dd, *J* = 13.7, 7.9 Hz, 1H) α-Gln, 4.28 (dd, *J* = 14.1, 9.6 Hz, 2H) α-Arg, α- Gln, 4.24 (m, 5H) α-His, α-Gln, α-Leu, α-Ser, α-Lys, 3.80 – 3.71 (m, 2H) α-Gly, β-Ser, 3.70 – 3.62 (m, 2H) α-Gly, β-Ser, 3.62 – 3.56 (m) δ-Arg, 3.09 (m) δ-Arg, ε-Lys, 3.03 – 2.98 (m, 1H) β-His, 2.94 – 2.89 (M, 1H) β-His, 2.76 -2.73 (m) ε-Lys, 2.12 (m) γ-Gln, 1.92 -1.82 (m) β-Gln, 1.76 – 1.71 (m) β-Gln, 1.69 – 1.64 (m) β-Arg, 1.60 (m, 2H) γ-Leu, 1.48 (m, 69H) β-Leu, γ-Arg, β-Lys, 1.30 (m, 2H) γ-Lys, 0.86 (d, *J* = 6.6 Hz, 3H) δ-Leu, 0.82 (d, *J* = 6.5 Hz, 3H) δ'-Leu. ^{13}C NMR (151 MHz, DMSO) δ 174.1 - 167.2 CO, 156.8 ε-Arg, 134.0 ε-His, 132.5 γ-His, 118.0 δ-His, 61.2 β-Ser, 54.7 α-His, 53.2 α-Lys, α-Arg, 52.2 α-Leu, 41.9 α-Gly, 41.0 β-Leu, 40.1 δ-Arg, 40.0 - 39.0 DMSO, 38.7 ε-Lys, 31.7 β-His, 31.3 γ-Gln, 27.6 β-Gln, β-Arg, 26.6 β-Lys, 24.8 γ-Arg, 24.1 γ-Leu, 23.0 δ-Leu, 22.1 γ-Lys, 21.4 δ'-Leu.

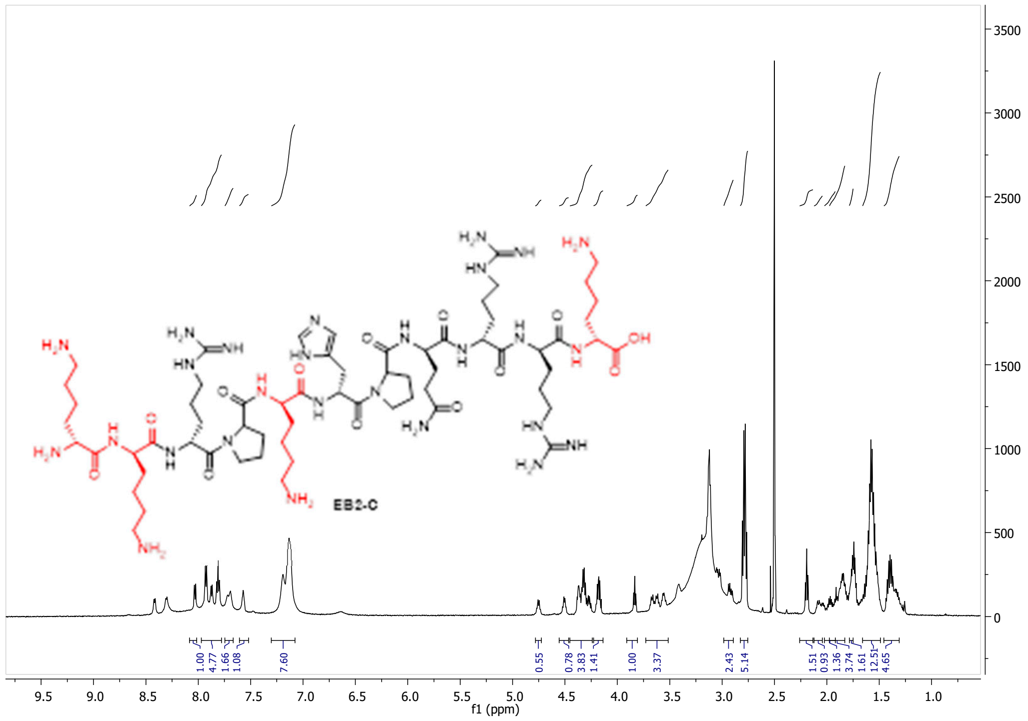

Figure S15. ^{1}H NMR spectrum of peptide **EB2-C** in DMSO-d_6

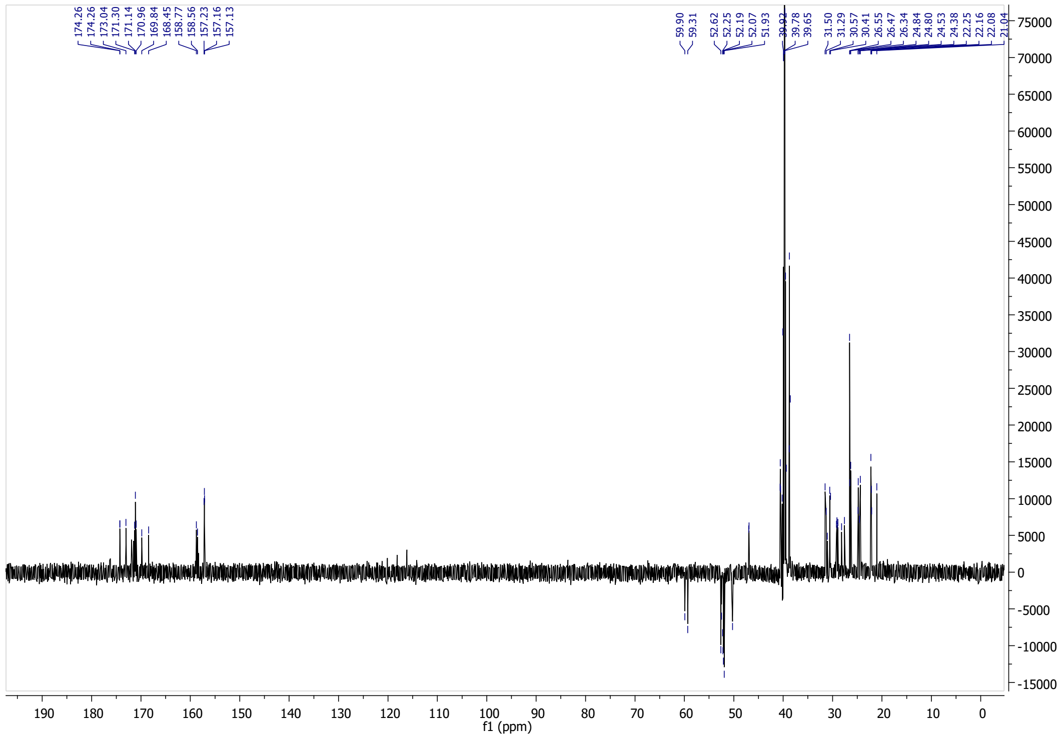

Figure S16. ^{13}H NMR spectrum of peptide **EB2-C** in DMSO-d_6

EB2-C ^{1}H NMR (600 MHz, DMSO) δ 8.42 - 7.14 NH, 4.75 (dd, *J* = 14.3, 7.5 Hz, 1H) α-Lys, 4.51 (dd, *J* = 12.6, 7.0 Hz, 1H) α-Arg, 4. 4.40 – 4.35 (m, 2H) α-Pro, 4.32 (dd, *J* = 13.4, 7.3 Hz, 3H) α-Lys, α-Gln, α-Arg, 4.27 (dd, *J* = 14.5, 9.0 Hz, 1H) α-Arg, 4.18 (td, *J* = 13.7, 8.3 Hz, 2H) α-Lys, α-His, 3.83 (t, *J* = 6.5 Hz, 1H) α-Lys, 3.69 – 3.64 (m, 1H) δ-Pro, 3.64 – 3.60 (m, 1H) δ-Pro, 3.60 – 3.53 (m, 1H) δ-Pro, 3.41 (m, 1H) δ-Pro, 3.13 (m) δ-Arg, 3.03 (m) ε-Lys, 2.92 (m) ε-Lys, 2.79 (m) ε-Lys, 2.19 (t, *J* = 7.6 Hz, 2H) γ-Gln, 2.12 – 2.02 (m, 2H) γ-Pro, 2.02 – 1.94 (m, 1H) γ-Pro, 1.91 (t, *J* = 6.9 Hz, 1H) β-Gln, 1.90 – 1.81 (m, 2H) β-Gln, γ-Pro, 1.76 -1.70 (m) δ-Lys, β-Arg, 1.66 – 1.48 (m) β-His, δ-Lys, β-Arg, γ-Arg, 1.37 (m) γ-Lys. ^{13}C NMR (151 MHz, DMSO) δ 174.3 - 168.5 CO, 157.2 ε-Arg, 157.2 ε-Arg, 157.1 ε-Arg, 59.9 α-Pro, 59.3 α-Pro, 52.6 α-Arg, 52.5 α-Arg, 52.3 α-Lys, 52.2 α-Gln, 52.1 α-Lys, 51.9 α-Lys, 50.2 α-Lys, 47.0 δ-Pro, 46.9 δ-Pro, 40.7 δ-Arg, 40.6 δ-Arg, 40.5 δ-Arg, 40.2 - 39.4 DMSO, 38.8 ε-Lys, 38.7 ε-Lys, 38.6 ε-Lys, 31.5 γ-Gln, 31.3 γ-Gln, 31.1 β-His 30.6 δ-Lys, 30.4 δ-Lys, 29.2 β-Arg, 29.1 β-β-Arg, 29.0 β-Gln, 28.9 β-Gln, 28.2 β-Pro, 27.5 β-Pro, 26.6 β-Lys, 26.5 β-Lys, 26.3 β-Lys, 24.8 γ-Arg, 24.8 γ-Arg, 24.5 γ-Pro, 24.4 γ-Pro, 22.3 γ-Lys, 22.2 γ-Lys, 22.1 γ-Lys, 21.0 γ-Lys.

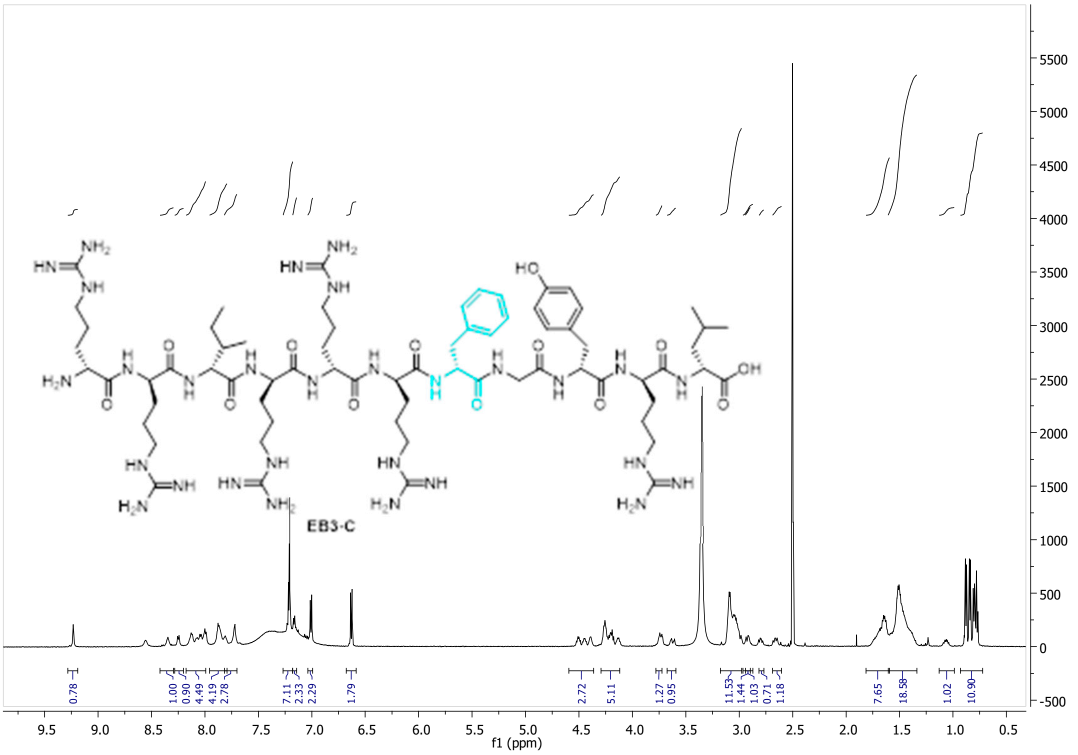

Figure S17. ^{1}H NMR spectrum of peptide **EB3-C** in DMSO-d_6

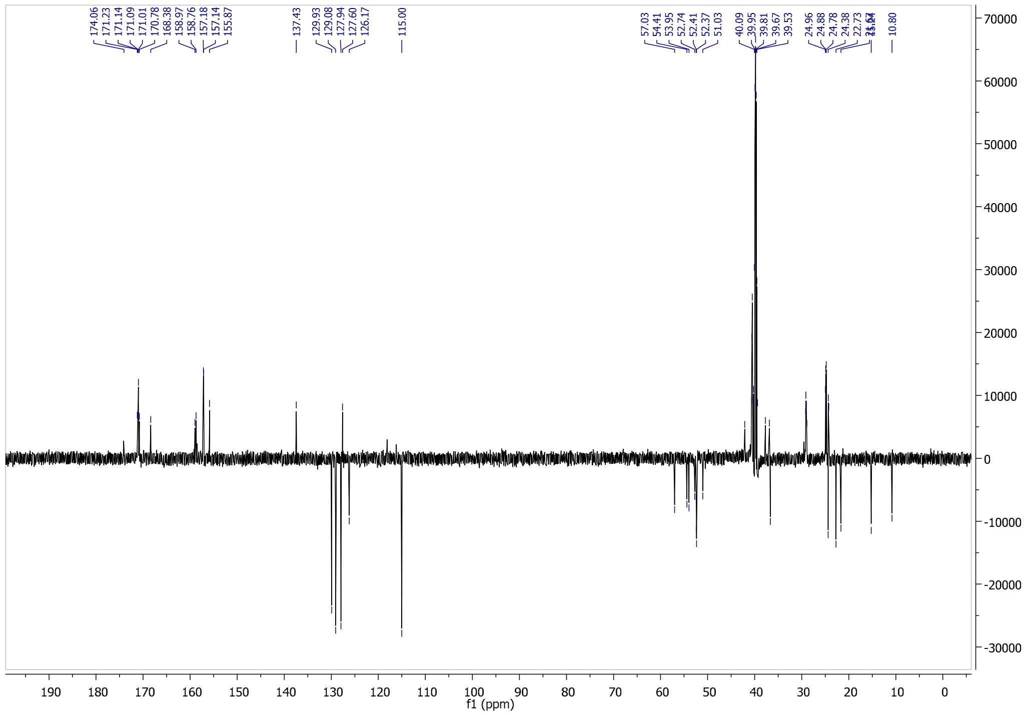

Figure S18. ^{13}H NMR spectrum of peptide **EB3-C** in DMSO-d_6

EB3-C ^{1}H NMR (600 MHz, DMSO) δ 8.97 - 7.57 NH, 7.24 - 7.20 (m) δ-Phe, ε-Phe, ζ-Phe, 7.18 - 7.02 NH, 7.01 - 7.00 (d, *J* = 8.6 Hz) δ-Tyr, 6.65 - 6.64 (d, *J* = 8.4 Hz) ε-Tyr, 4.57 - 4.53 (m) α-Arg, 4.51 - 4.48 (m) α-Arg, 4.40 - 4.38 (m) α-Arg, 4.32 - 4.26 (m) α-Tyr, 4.25 - 4.20 (m) α-Leu, α-ILe, 3.75 - 3.63 (m) α-Gly, 3.14 - 3.02 (m) δ-Arg, β-Phe, 2.97 - 2.94 (m) β-Phe, 2.87 - 2.83 (m) β-Tyr, 2.74 - 2.70 (m) β-Tyr, 2.51 - 2.49 DMSO, 1.79 - 1.43 (m) β-Ile, β-Leu, γ-Ile, γ-Leu, γ-Arg, 0.92 - 0.81 (m) δ-Leu, δ-Ile, CH_3-(β-Ile). ^{13}C NMR (151 MHz, DMSO) δ 174.1 - 168.4 CO, 159.0 ε-Arg, 158.8 ε-Arg, 157.2 ε-Arg, 157.1 ε-Arg, 155.9 ξ-Tyr, 137.4 γ-Phe, 129.9 δ-Tyr, 129.1 ε-Phe, 127.9 δ-Tyr, 127.6 γ-Tyr, 126.2 ζ-Phe, 115.0 ε-Tyr, 57.0 α-Ile, 54.4 α-Phe, 54.0 α-Tyr, 52.7 α-Arg, 52.4 α-Arg, 51.0 α-Arg, 42.1 α-Gly, 40.7 δ-Arg, 40.6 δ-Arg, 40.5 δ-Arg, 40.5 δ-Arg, 40.2 - 39.4 DMSO, 37.7 β-Phe, 36.9 β-Tyr β, 36.7 β-Ile, 29.2 β-Arg, 29.1 β-Arg , 29.0 β-Arg , 28.9 β-Arg, 25.0 γ-Arg, 24.9 γ-Arg, 24.8 γ-Arg , 24.4 γ-Arg , 24.3 γ-Arg , 24.3 γ-Leu, 22.7 δ-Leu, 21.7 δ-Leu, 15.2 β-Ile, 10.8 δ-Ile.

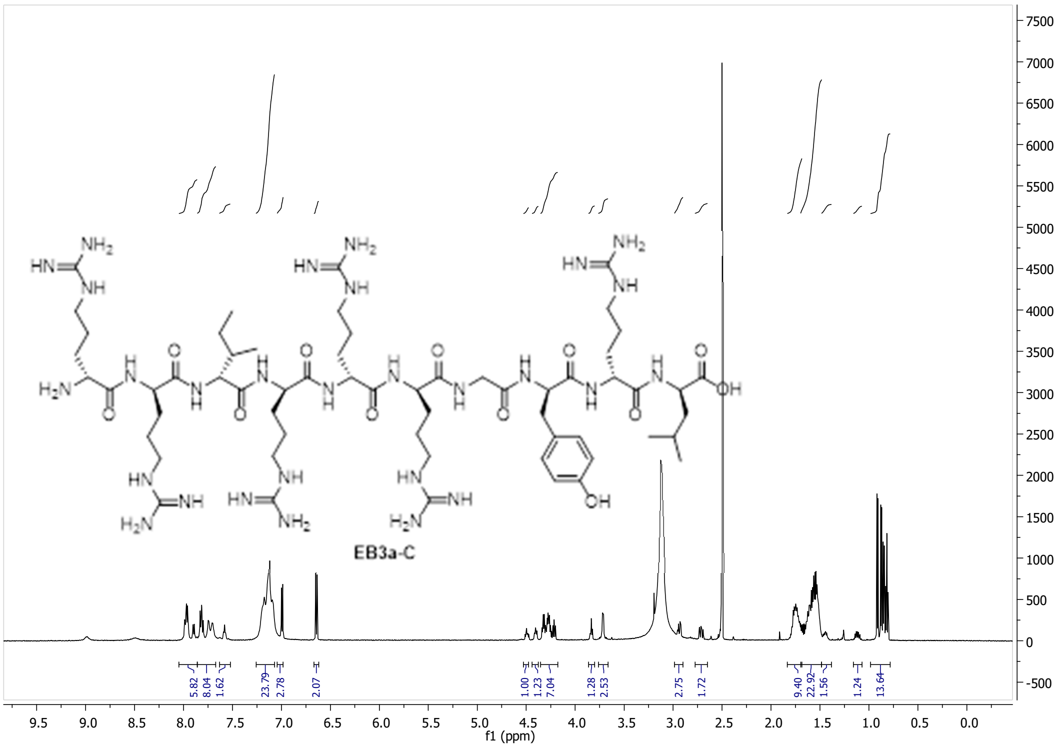

Figure S19. ^{1}H NMR spectrum of peptide **EB3a-C** in DMSO-d_6

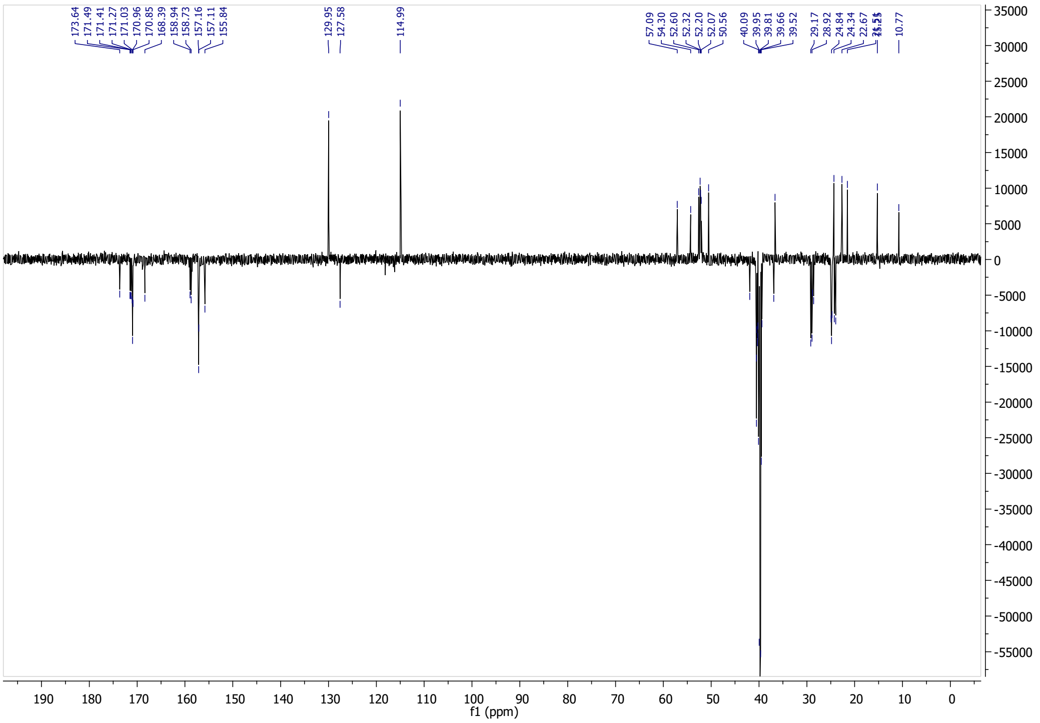

Figure S20. ^{13}H NMR spectrum of peptide **EB3a-C** in DMSO-d_6

EB3a-C ^{1}H NMR (600 MHz, DMSO) δ 8.02 – 7.58 NH, 7.32 – 7.04 (m) NH, 6.99 (d, *J* = 8.5 Hz, 2H) δ-Tyr, 6.68 – 6.62 (d, *J* = 8.5 Hz, 2H) ε-Tyr, 4.49 (dd, *J* = 13.2, 8.3 Hz, 1H) α-Tyr, 4.43 – 4.38 (m, 1H) α-Leu, 4.32 (dd, *J* = 13.8, 8.0 Hz, 2H) α-Arg, 4.30 – 4.25 (m, 3H) α-Arg, 4.22 (dd, *J* = 15.3, 7.6 Hz, 1H) α-Ile, 3.83 (t, *J* = 6.4 Hz, 1H) α-Arg, 3.72 (d, *J* = 6.8 Hz, 2H) α-Gly, 2.94 (dd, *J* = 14.1, 4.7 Hz, 2H) β-Tyr, 2.71 (dd, *J* = 14.1, 8.7 Hz, 1H) β-Tyr, 1.72 (ddd, *J* = 19.9, 14.6, 6.5 Hz, 9H) β-Ile, β-Arg 1.67 – 1.48 (m) β-Arg, γ-Leu 1.45 (ddd, *J* = 13.4, 7.5, 3.5 Hz, 2H) β-Leu, 1.16 – 1.08 (m, 1H) γ-Ile , 0.91 (d, *J* = 6.6 Hz, 3H) δ-Leu, 0.87 (d, *J* = 6.5 Hz, 3H) δ'-Leu, 0.85 (d, *J* = 6.8 Hz, 3H) CH_3-(β-Ile), 0.82 (t, *J* = 7.4 Hz, 3H) ε-Ile. ^{13}C NMR (151 MHz, DMSO) δ 173.6 - 168.4 CO, 157.2 ε-Arg, 157.1 β-Arg, 155.8 ζ-Tyr, 120,0 δ-Tyr, 127.6 γ-Tyr, 115.0 ε-Tyr, 57.1 α-Ile, 54.3 α-Tyr, 52.6 α-Arg, 52.3 α-Arg, 52.2 α-Arg, 52.1 α-Arg, 50.6 α-Leu, 41.9 α-Gly, 40.6 δ-Arg, 40.5 β-Leu, 40.5 δ-Arg, 40.3 δ-Arg, 40.3 δ-Arg, 40.2 δ-Arg, 40.0 - 39.4 DMSO, 36.9 β-Tyr, 36.6 β-Ile, 29.2 β-Arg, 28.9 β-Arg, 28.8 β-Arg, 28.6 β-Arg, 24.9 γ-Arg, 24.8 γ-Arg, 24.7 γ-Arg, 24.3 γ-Ile, 24.3 γ-Arg, 24.0 γ-Ile, 22.7 δ-Leu, 21.5 δ'-Leu, 15.3 CH_3-(β-Ile) , 10.8 ε-Ile.

3. MS analysis of peptide EB derivatives

ID 1: EB-1

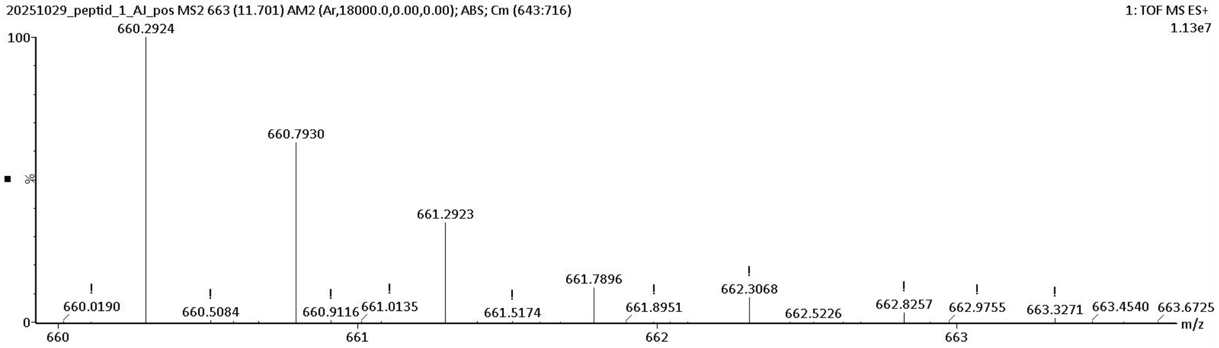

Figure S21. Zoomed-in mass spectrum showing isotope distribution of the doubly charged $[M+2H]^{2+}$ ion. Theoretical *m/z* 660.2925; observed *m/z* 660.2924.

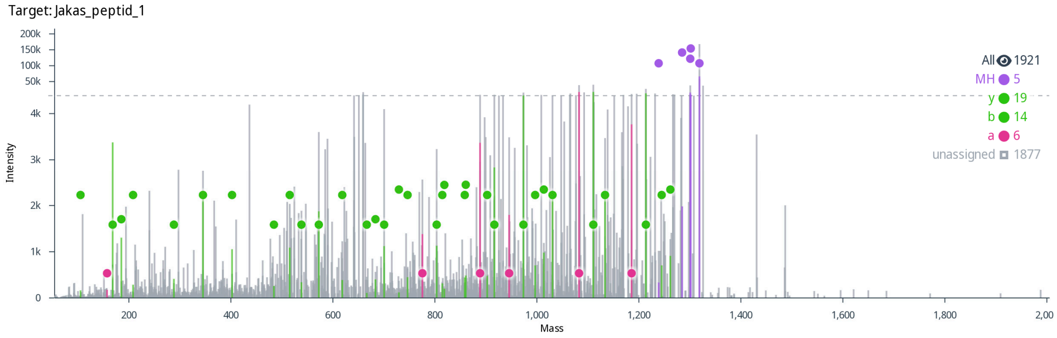

Figure S22. Mass spectrum of GQCQRKCLGHCS peptide acquired in MS/MS mode on Synapt G2-Si as analyzed by ExDViewer software.

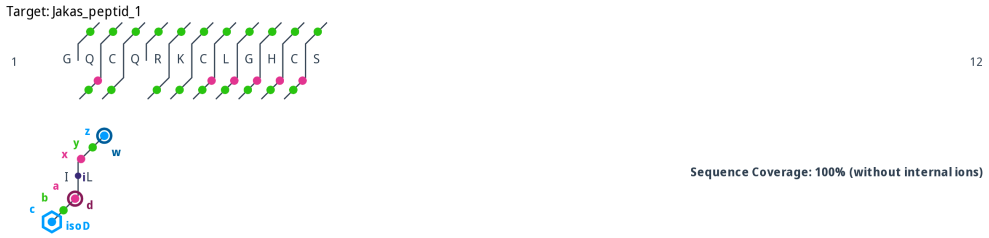

Figure S23. Sequence coverage for GQCQRKCLGHCS peptide calculated by ExDViewer software.

Table S2. Summary of MS/MS identification metrics for GQCQRKCLGHCS obtained by ExDViewer software.

	Index	Target	Sequence	# Ions	# Deconv Ions	Total Ion Score	Precursor Ion Score	Fragmentation Ion Score	RT Start (sec)	RT End (sec)	M/Z Error	M/Z	Z	# Spectra
✓	0	Jakas_peptid_1	.GQCQRKCLGHCS.	60	44	251.7	22.9	228.9	691.800	691.800	-0.0005360	660.2925	40	1

ID 2: EB-2

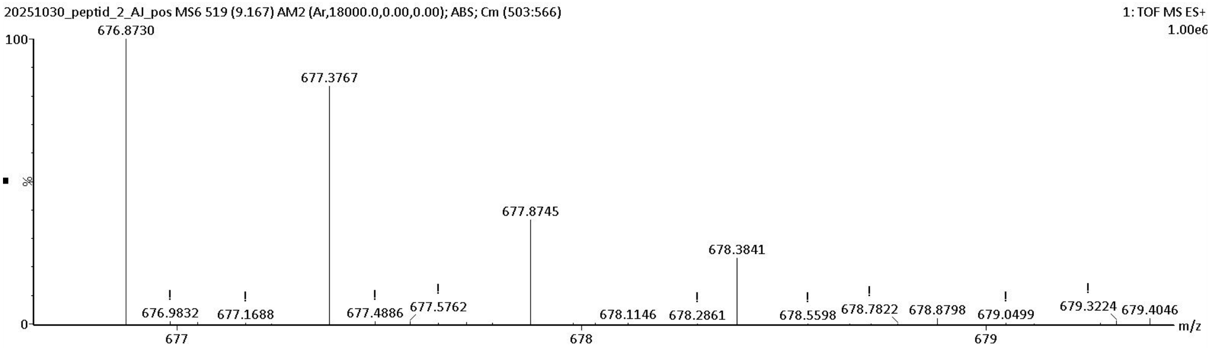

Figure S24. Zoomed-in mass spectrum showing isotope distribution of the doubly charged $[M+2H]^{2+}$ ion. Theoretical *m/z* 676.8743; observed *m/z* 676.8730.

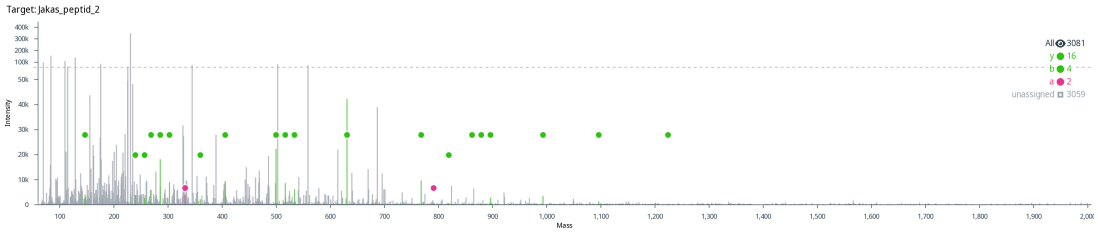

Figure S25. Mass spectrum of KKCPKHPQCRK peptide acquired in MS/MS mode on Synapt G2-Si as analyzed by ExDViewer software.

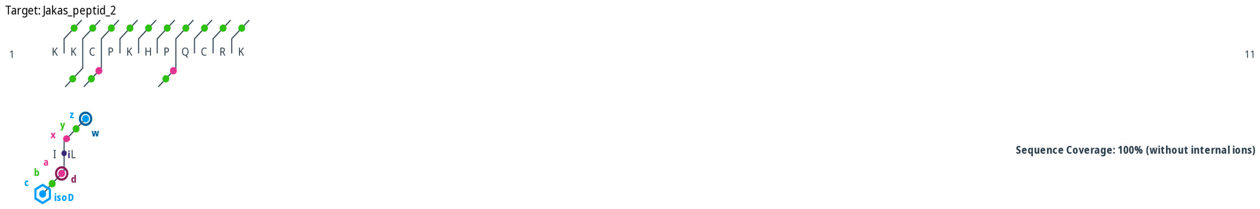

Figure S26. Sequence coverage for KKCPKHPQCRK peptide calculated by ExDViewer software.

Table S3. Summary of MS/MS identification metrics for KKCPKHPQCRK obtained by ExDViewer software.

	Index	Target	Sequence	# Ions	# Deconv Ions	Total Ion Score	Precursor Ion Score	Fragmentation Ion Score	RT Start (sec)	RT End (sec)	M/Z Error	M/Z	Z	# Spectra
✓	0	Jakas_peptid_2	.KKCPKHPQCRK.	25	22	221.7	0.0	221.7	550.200	550.200	-0.0005971	676.8743	40	1

ID 3: EB-3

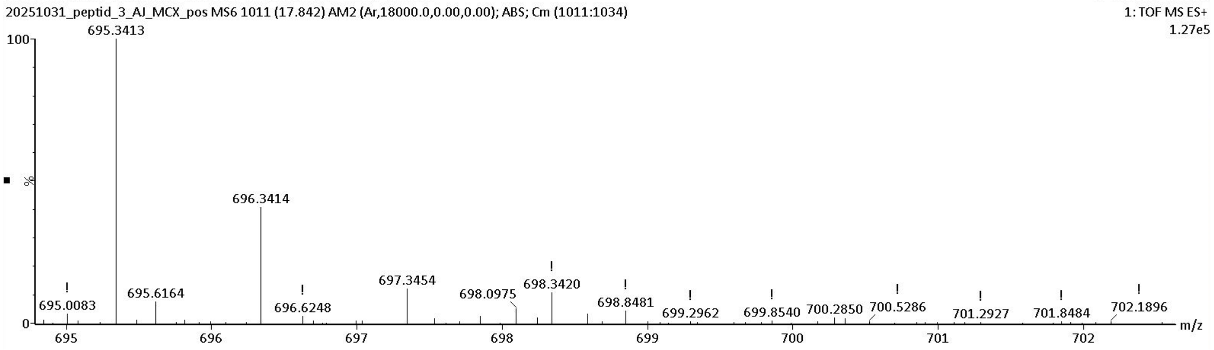

Figure S27. Zoomed-in mass spectrum showing isotope distribution of the doubly charged $[M+2H]^{2+}$ ion. Theoretical *m/z* 695.3392; observed *m/z* 695.3413.

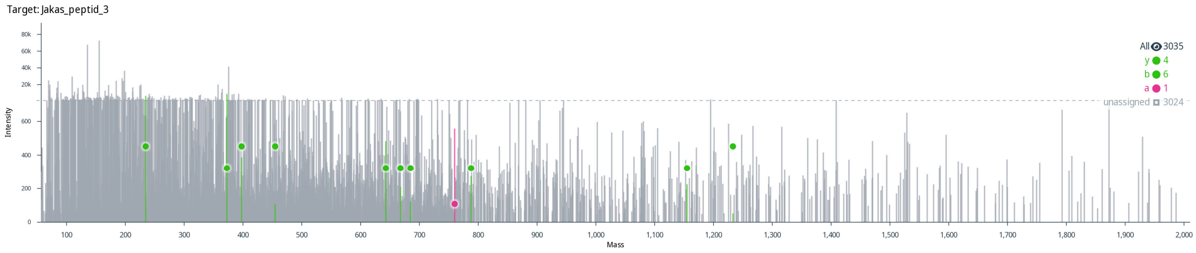

Figure S28. Mass spectrum of RCIRRCFGYCL peptide acquired in MS/MS mode on Synapt G2-Si as analyzed by ExDViewer software.

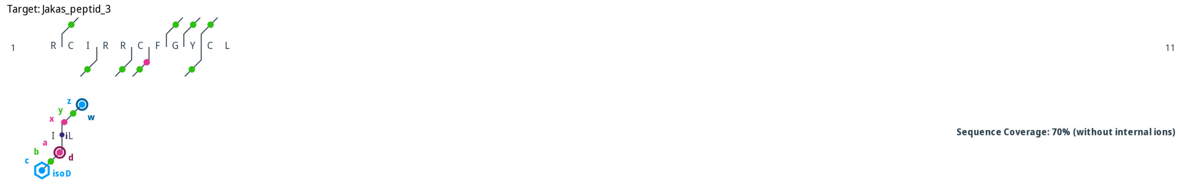

Figure S29 Sequence coverage for RCIRRCFGYCL peptide calculated by ExDViewer software.

Table S4. Summary of MS/MS identification metrics for RCIRRCFGYCL obtained by ExDViewer software.

	Index	Target	Sequence	# Ions	# Deconv Ions	Total Ion Score	Precursor Ion Score	Fragmentation Ion Score	RT Start (sec)	RT End (sec)	M/Z Error	M/Z	Z	# Spectra
✓	0	Jakas_peptid_3	.RCIRRCFGYCL.	12	11	49.9	0.0	49.9	1083.600	1083.600	-0.0004750	695.3392	40	1

ID 4: EB1a-K

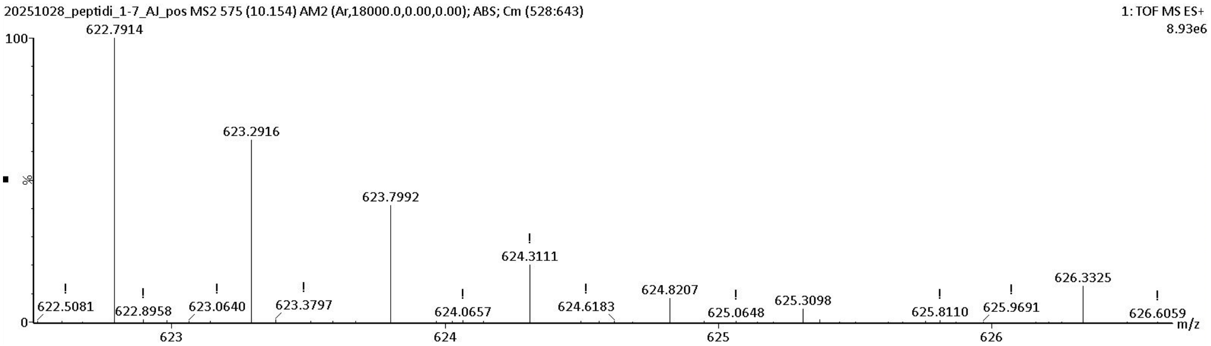

Figure S30. Zoomed-in mass spectrum showing isotope distribution of the doubly charged $[M+2H]^{2+}$ ion. Theoretical *m/z* 622.7909; observed *m/z* 622.7914.

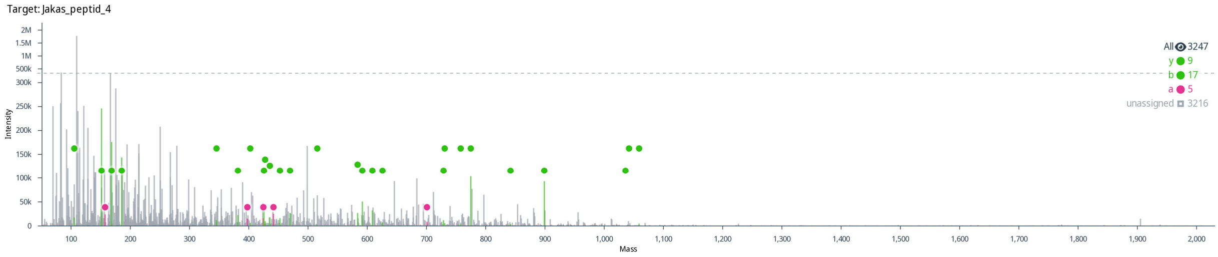

Figure S31. Mass spectrum of GQQRRCLGHCS peptide acquired in MS/MS mode on Synapt G2-Si as analyzed by ExDViewer software.

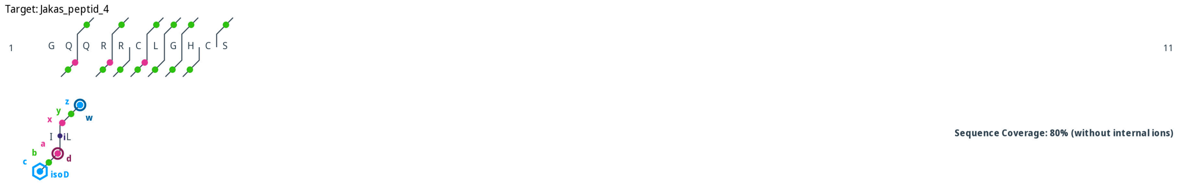

Figure S32. Sequence coverage for GQQRRCLGHCS peptide calculated by ExDViewer software.

Table S5. Summary of MS/MS identification metrics for GQQRRCLGHCS obtained by ExDViewer software.

	Index	Target	Sequence	# Ions	# Deconv Ions	Total Ion Score	Precursor Ion Score	Fragmentation Ion Score	RT Start (sec)	RT End (sec)	M/Z Error	M/Z	Z	# Spectra
✓	0	Jakas_peptid_4	.GQQRRCLGHCS.	35	31	227.6	0.0	227.6	610.200	610.200	-0.0004750	622.7909	40	1

ID 5: EB1-K

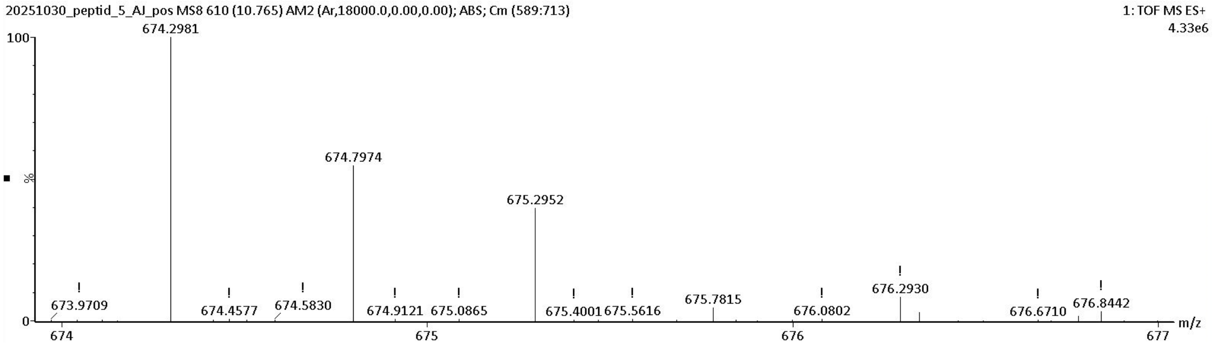

Figure S33. Zoomed-in mass spectrum showing isotope distribution of the doubly charged $[M+2H]^{2+}$ ion. Theoretical *m/z* 674.2955; observed *m/z* 674.2981.

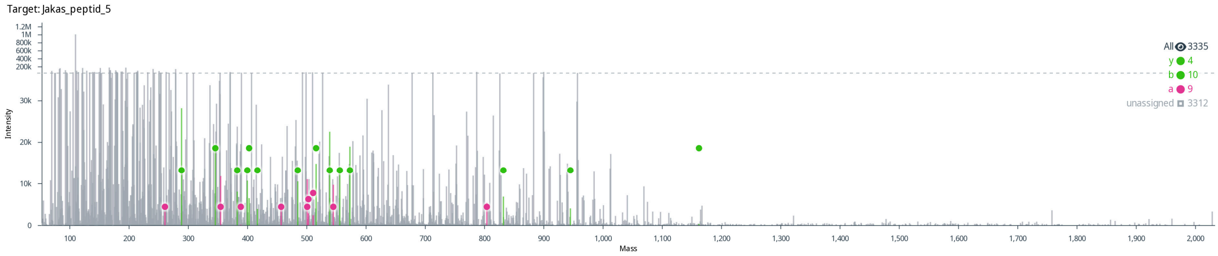

Figure S34. Mass spectrum of GQCQRRCLGHCS peptide acquired in MS/MS mode on Synapt G2-Si as analyzed by ExDViewer software.

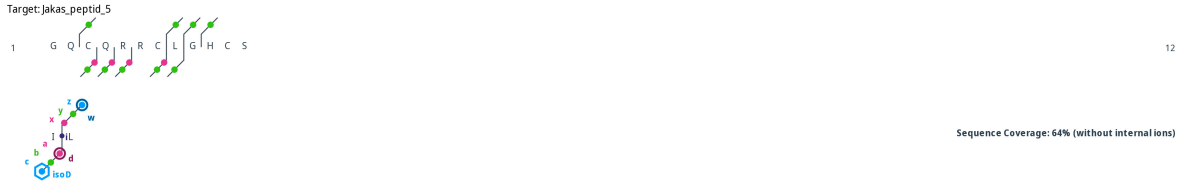

Figure S35. Sequence coverage for GQCQRRCLGHCS peptide calculated by ExDViewer software.

Table S6. Summary of MS/MS identification metrics for GQCQRRCLGHCS obtained by ExDViewer software.

	Index	Target	Sequence	# Ions	# Deconv Ions	Total Ion Score	Precursor Ion Score	Fragmentation Ion Score	RT Start (sec)	RT End (sec)	M/Z Error	M/Z	Z	# Spectra
✓	0	Jakas_peptid_5	.GQCQRRCLGHCS.	24	23	189.2	0.0	189.2	646.200	646.200	-0.0004750	674.2955	40	1

ID 6: EB2-K

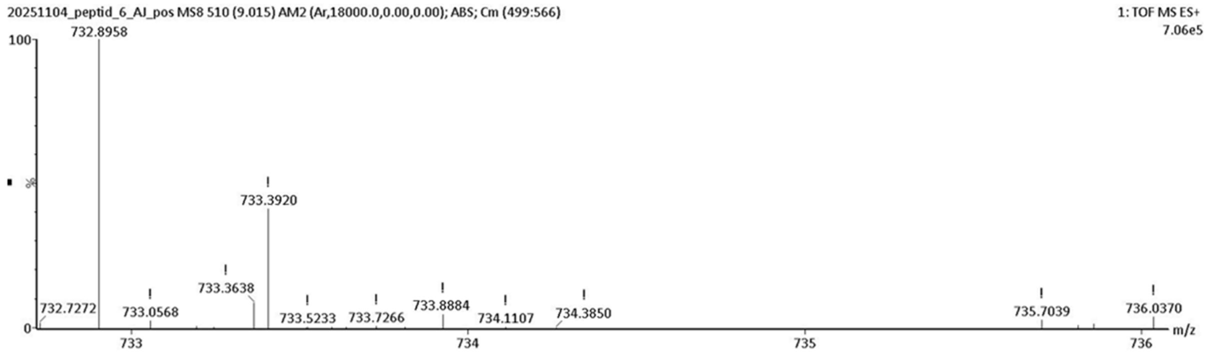

Figure S36. Zoomed-in mass spectrum showing isotope distribution of the doubly charged $[M+2H]^{2+}$ ion. Theoretical *m/z* 732.8866; observed *m/z* 732.8958.

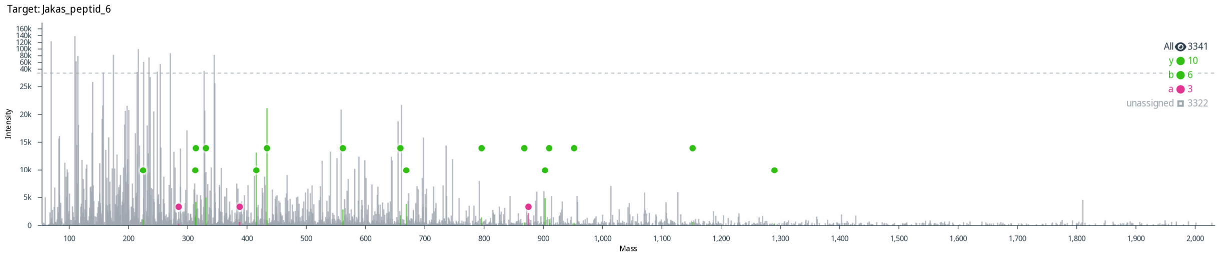

Figure S37. Mass spectrum of RRCPRHPQCRR peptide acquired in MS/MS mode on Synapt G2-Si as analyzed by ExDViewer software.

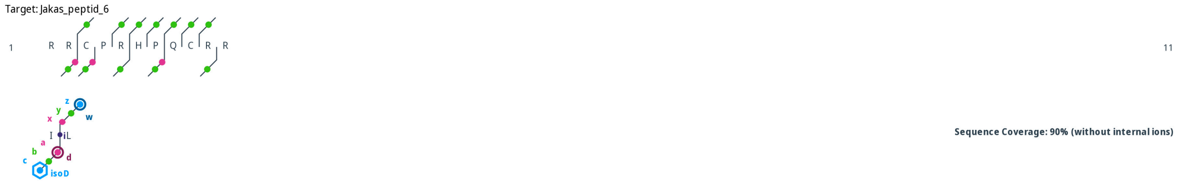

Figure S38. Sequence coverage for RRCPRHPQCRR peptide calculated by ExDViewer software.

Table S7. Summary of MS/MS identification metrics for RRCPRHPQCRR obtained by ExDViewer software.

	Index	Target	Sequence	# Ions	# Deconv Ions	Total Ion Score	Precursor Ion Score	Fragmentation Ion Score	RT Start (sec)	RT End (sec)	M/Z Error	M/Z	Z	# Spectra
✓	0	Jakas_peptid_6	.RRCPRHPQCRR.	22	19	106.5	0.0	106.5	550.200	550.200	-0.0005360	732.8866	40	1

ID 7: EB1-C

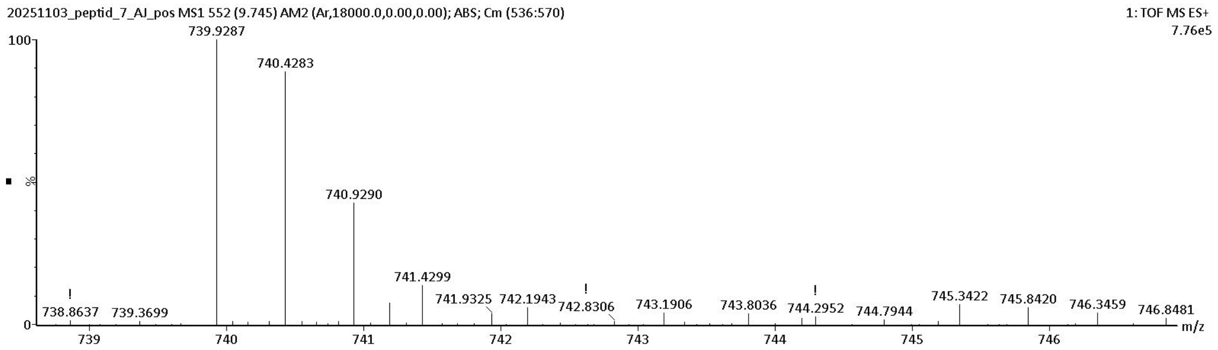

Figure S39. Zoomed-in mass spectrum showing isotope distribution of the doubly charged $[M+2H]^{2+}$ ion. Theoretical *m/z* 739.9304; observed *m/z* 739.9287.

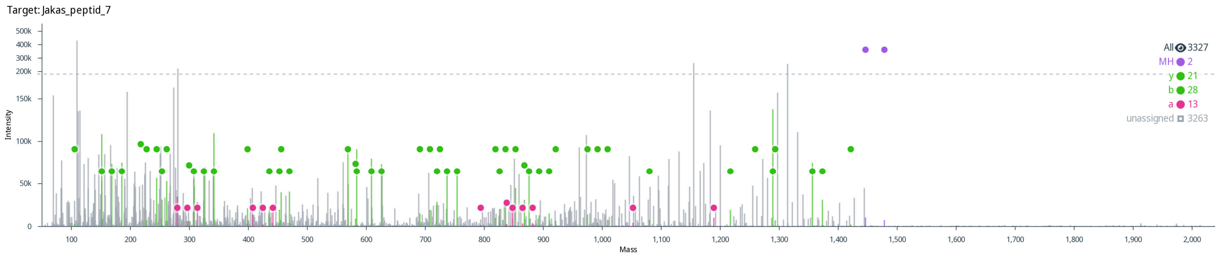

Figure S40. Mass spectrum of GQRQRKRLGHRS peptide acquired in MS/MS mode on Synapt G2-Si as analyzed by ExDViewer software.

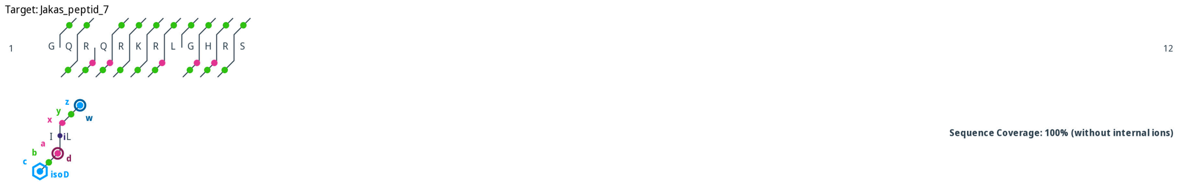

Figure S41. Sequence coverage for GQRQRKRLGHRS peptide calculated by ExDViewer software.

Table S8. Summary of MS/MS identification metrics for GQRQRKRLGHRS obtained by ExDViewer software.

	Index	Target	Sequence	# Ions	# Deconv Ions	Total Ion Score	Precursor Ion Score	Fragmentation Ion Score	RT Start (sec)	RT End (sec)	M/Z Error	M/Z	Z	# Spectra
✓	0	Jakas_peptid_7	.GQRQRKRLGHRS.	72	64	640.1	21.8	618.3	585.600	585.600	-0.0005971	739.9304	40	1

ID 8: EB2-C

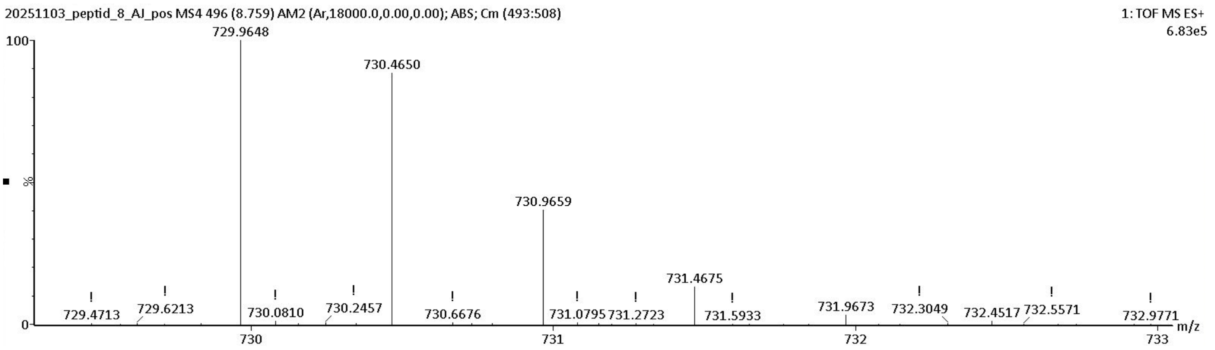

Figure S42. Zoomed-in mass spectrum showing isotope distribution of the doubly charged $[M+2H]^{2+}$ ion. Theoretical *m/z* 729.9662; observed *m/z* 729.9648.

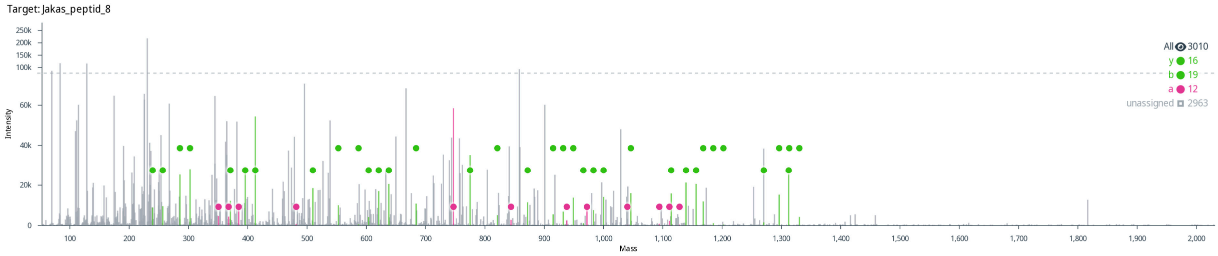

Figure S43. Mass spectrum of KKRPKHPQRRK peptide acquired in MS/MS mode on Synapt G2-Si as analyzed by ExDViewer software.

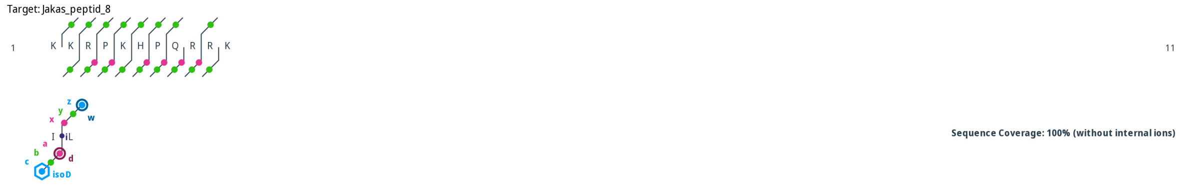

Figure S44. Sequence coverage for KKRPKHPQRRK peptide calculated by ExDViewer software.

Table S9. Summary of MS/MS identification metrics for KKRPKHPQRRK obtained by ExDViewer software.

	Index	Target	Sequence	# Ions	# Deconv Ions	Total Ion Score	Precursor Ion Score	Fragmentation Ion Score	RT Start (sec)	RT End (sec)	M/Z Error	M/Z	Z	# Spectra
✓	0	Jakas_peptid_8	.KKRPKHPQRRK.	63	47	602.5	0.0	602.5	525.600	525.600	-0.0005360	729.9662	40	1

ID 9: EB3-C

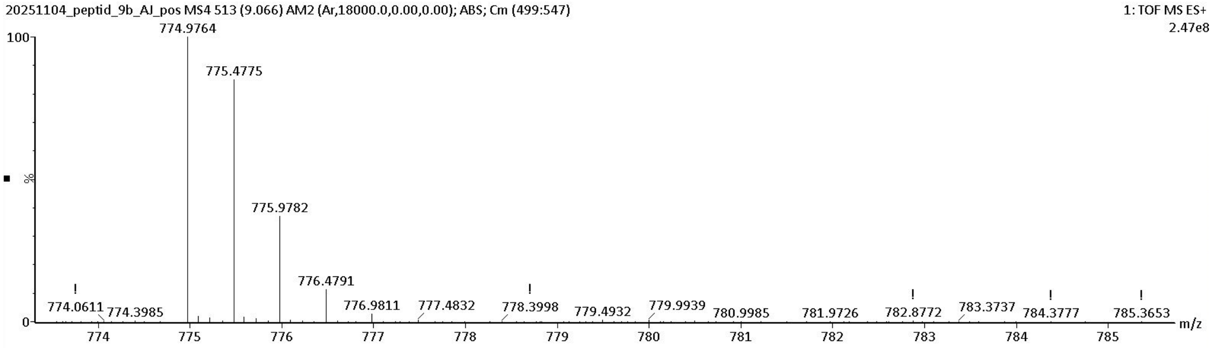

Figure S45. Zoomed-in mass spectrum showing isotope distribution of the doubly charged $[M+2H]^{2+}$ ion. Theoretical *m/z* 774.9771; observed *m/z* 774.9764.

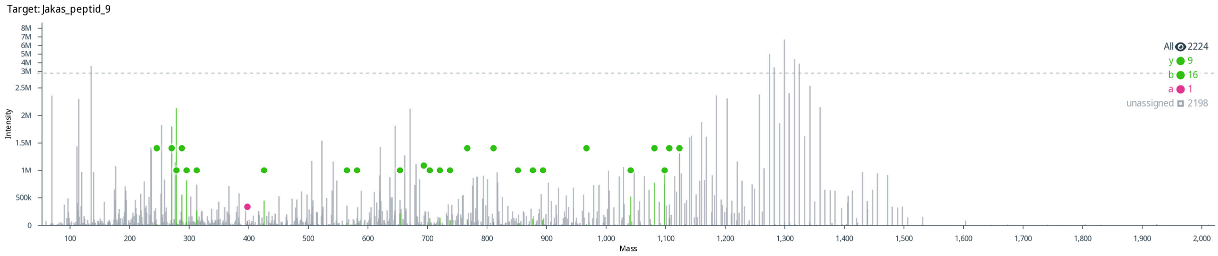

Figure S46. Mass spectrum of RRIRRRFGYRL peptide acquired in MS/MS mode on Synapt G2-Si as analyzed by ExDViewer software.

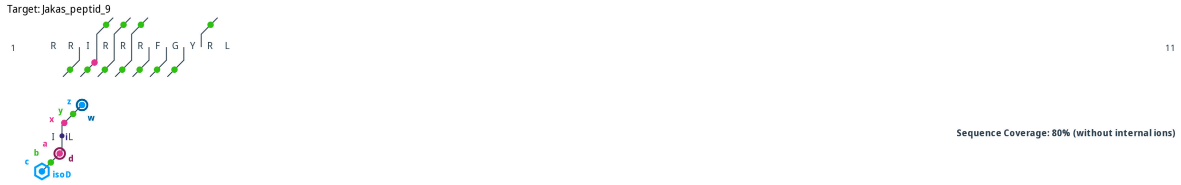

Figure S47. Sequence coverage for RRIRRRFGYRL peptide calculated by ExDViewer software.

Table S10. Summary of MS/MS identification metrics for RRIRRRFGYRL obtained by ExDViewer software.

	Index	Target	Sequence	# Ions	# Deconv Ions	Total Ion Score	Precursor Ion Score	Fragmentation Ion Score	RT Start (sec)	RT End (sec)	M/Z Error	M/Z	Z	# Spectra
✓	0	Jakas_peptid_9	.RRIRRRFGYRL.	32	26	290.4	0.0	290.4	544.200	544.200	-0.0005360	774.9771	40	1

ID 10: EB3a-C

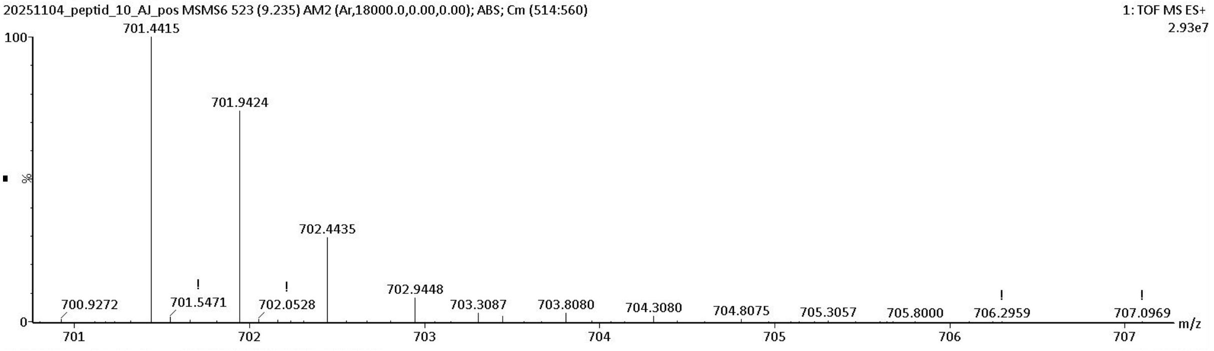

Figure S48. Zoomed-in mass spectrum showing isotope distribution of the doubly charged $[M+2H]^{2+}$ ion. Theoretical *m/z* 701.4429; observed *m/z* 701.4415.

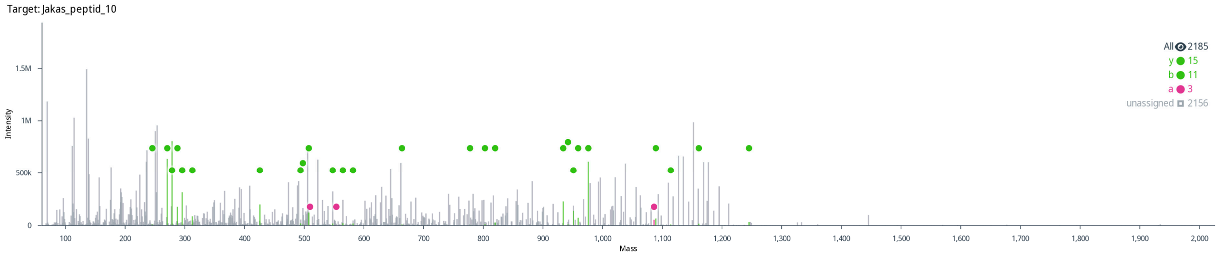

Figure S49. Mass spectrum of RRIRRRGYRL peptide acquired in MS/MS mode on Synapt G2-Si as analyzed by ExDViewer software.

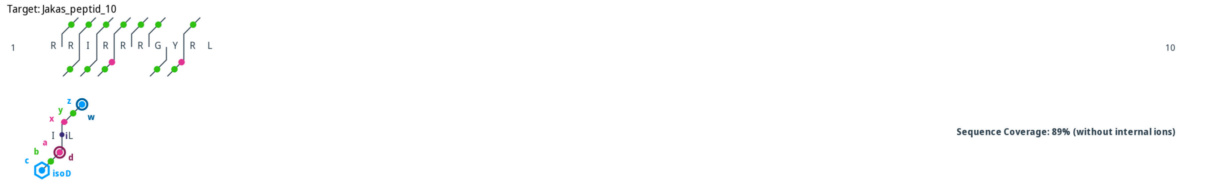

Figure S50. Sequence coverage for RRIRRRGYRL peptide calculated by ExDViewer software.

Table S11. Summary of MS/MS identification metrics for RRIRRRGYRL obtained by ExDViewer software.

	Index	Target	Sequence	# Ions	# Deconv Ions	Total Ion Score	Precursor Ion Score	Fragmentation Ion Score	RT Start (sec)	RT End (sec)	M/Z Error	M/Z	Z	# Spectra
✓	0	Jakas_peptid_10	.RRIRRRGYRL.	31	29	268.6	0.0	268.6	553.800	553.800	-0.0005360	701.4429	40	1

4. MS analysis of peptides for disulfide bond detection

Peptide ID 1: **EB1**

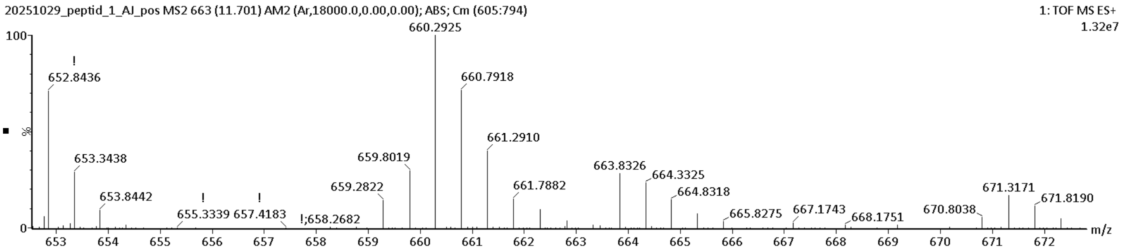

Figure S51. Zoomed-in mass spectrum of the doubly charged $[M+2H]^{2+}$ ion showing the reduced form (m/z 660.2925) and a minor oxidized species (m/z 659.2846) consistent with one intramolecular disulfide bond.

Peptide ID 2: **EB2**

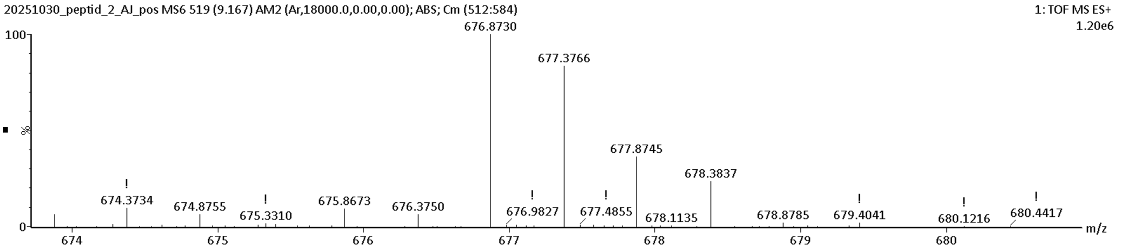

Figure S52. Zoomed-in mass spectrum of the doubly charged $[M+2H]^{2+}$ ion showing the reduced form (m/z 676.8730) and a minor oxidized species (m/z 675.8673) consistent with one intramolecular disulfide bond.

Peptide ID 4: **EB1a-K**

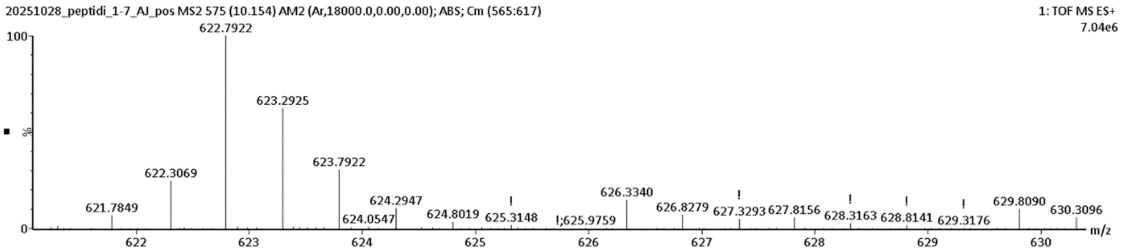

Figure S53. Zoomed-in mass spectrum of the doubly charged $[M+2H]^{2+}$ ion showing the reduced form (m/z 622.7922) and a minor oxidized species (m/z 621.7849) consistent with one intramolecular disulfide bond.

Peptide ID 5: EB2-K

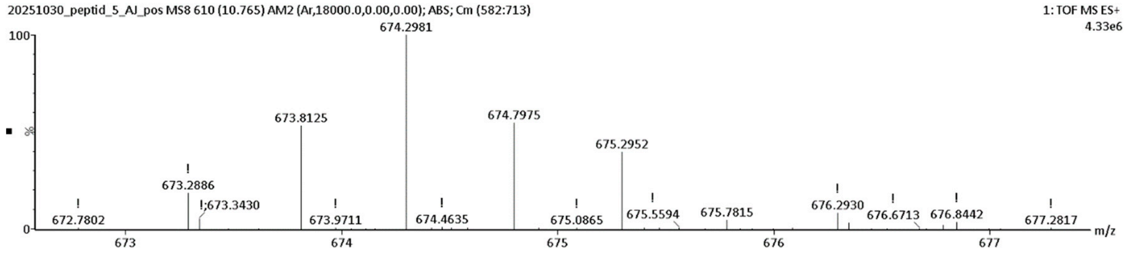

Figure S54. Zoomed-in mass spectrum of the doubly charged $[M+2H]^{2+}$ ion showing the reduced form (m/z 674.2981.7922) and a minor oxidized species (m/z 673.2886) consistent with one intramolecular disulfide bond.
